# ELAVL2 loss promotes aggressive mesenchymal transition in glioblastoma

**DOI:** 10.1038/s41698-024-00566-1

**Published:** 2024-03-28

**Authors:** Yona Kim, Ji Hyeon You, Yeonjoo Ryu, Gyuri Park, Urim Lee, Hyo Eun Moon, Hye Ran Park, Chang W. Song, Ja-Lok Ku, Sung-Hye Park, Sun Ha Paek

**Affiliations:** 1 Department of Neurosurgery, Cancer Research Institute and Ischemic/Hypoxic Disease Institute, Seoul National University College of Medicine, Seoul, Korea; 2 Interdisciplinary Program in Neuroscience, Seoul National University College of Biological Sciences, Seoul, Korea; 3 Interdisciplinary Program in Caner Biology, Seoul National University College of Medicine, Seoul, Korea; 4 Department of Neurosurgery, Soonchunhyang University Seoul Hospital, Seoul, Korea; 5 Department of Radiation Oncology, University of Minnesota Medical School, Minneapolis, MN 55455 USA; 6 Korean Cell Line Bank, Laboratory of Cell Biology, Cancer Research Institute, Seoul National University College of Medicine, Seoul, Korea; 7 Department of Pathology, Seoul National University Hospital, Seoul, Korea; 8 Advanced Institute of Convergence Technology, Seoul National University, Suwon, Korea

**Keywords:** CNS cancer, Prognostic markers, CNS cancer

## Abstract

Glioblastoma (GBM), the most lethal primary brain cancer, exhibits intratumoral heterogeneity and molecular plasticity, posing challenges for effective treatment. Despite this, the regulatory mechanisms underlying such plasticity, particularly mesenchymal (MES) transition, remain poorly understood. In this study, we elucidate the role of the RNA-binding protein ELAVL2 in regulating aggressive MES transformation in GBM. We found that ELAVL2 is most frequently deleted in GBM compared to other cancers and associated with distinct clinical and molecular features. Transcriptomic analysis revealed that ELAVL2-mediated alterations correspond to specific GBM subtype signatures. Notably, ELAVL2 expression negatively correlated with epithelial-to-mesenchymal transition (EMT)-related genes, and its loss promoted MES process and chemo-resistance in GBM cells, whereas ELAVL2 overexpression exerted the opposite effect. Further investigation via tissue microarray analysis demonstrated that high ELAVL2 protein expression confers a favorable survival outcome in GBM patients. Mechanistically, ELAVL2 was shown to directly bind to the transcripts of EMT-inhibitory molecules, SH3GL3 and DNM3, modulating their mRNA stability, potentially through an m6A-dependent mechanism. In summary, our findings identify ELAVL2 as a critical tumor suppressor and mRNA stabilizer that regulates MES transition in GBM, underscoring its role in transcriptomic plasticity and glioma progression.

## Introduction

Glioblastoma (GBM) is a deadly primary malignancy of the central nervous system in adults^1^, and the current therapeutic approach includes surgery, adjuvant radiotherapy, and chemotherapy with temozolomide (TMZ), an alkylating agent^2^. GBM has been found to be highly heterogeneous at both inter- and intratumoral levels, and such a molecular complexity has been shown to contribute to multi-therapy resistance and tumor recurrence^3^. Large-scale transcriptomic analysis of GBM has identified three major signatures - proneural (PN), classical, and mesenchymal (MES) - in association with the oncogenic pathways and genomic alterations^4,5^. The transitions between subtypes, especially that of proneural-to-mesenchymal transition (PMT), have been identified as a major contributing factor to intratumoral complexity and heterogeneity^1,6–8^. The PMT has been considered the equivalent to epithelial-to-mesenchymal transition (EMT), a well-established factor driving aggressiveness in carcinomas^9^. However, the molecular basis underlying GBM plasticity in relation to MES transition is still not fully understood. Therefore, identifying a novel prognostic marker that regulates such plasticity is critical to unravel the complex molecular heterogeneity of GBM and develop effective therapies.

RNA-binding proteins (RBPs) recognize and bind to specific sequences of RNA through one or multiple RNA‐binding domains in a spatiotemporal regulatory manner^10^. They form ribonucleoprotein complexes and maintain the transcriptome by post-transcriptionally regulating the processing and transportation of RNA. Abnormal alterations in this process affect the RNA life cycle and produce abnormal gene products^11^. Also, RBPs were shown to control several aspects of RNA modifications, including m^6^A methylation, RNA splicing, polyadenylation, mRNA stability, and mRNA localization^12,13^. Accumulating evidence indicates that aberrant expression mechanisms of RBPs and RBP-mediated RNA modifications are critical for cancer development and progression^14^.

ELAV (embryonic lethal and abnormal vision), initially discovered in *Drosophila*, is a highly conserved RBP, whose molecular function was reported to control differentiation, development, and maintenance of neural cells or related cell types across species^15,16^. ELAV RBPs recognize poly-U elements or AU-rich elements in the 3′-untranslated regions of target gene transcripts, thereby regulating gene expression at the post-transcriptional level^17^. Mechanistically, most ELAV proteins prevent exonuclease- or endonuclease-mediated degradation of target mRNA transcripts by hindering the association between ARE-containing transcripts and other ARE-binding RBPs, thereby stabilizing these transcripts^18^. In humans, the ELAVL (ELAV-like) RBP gene family consists of four members – the ubiquitously expressed ELAVL1 (HuR) and the primarily neuronal RBPs ELAVL2, 3, and 4 (HuB, HuC, and HuD)^19^. These neuronal RBPs have been reported to act as neuron-specific alternative splicing regulators and also control several target transcripts important in synaptic plasticity, axonogenesis, and memory-related neurotransmission pathways^19,20^.

Interestingly, Elavl2 was found to be differentially expressed and regulated from other neuronal Elavl members, being expressed in a region- and cell type-specific manner in the mouse hippocampus^21^. Also, ELAVL2 has been shown to play pivotal roles in regulating a variety of biological processes, ranging from neuronal differentiation, memory consolidation to the formation of primordial follicles and the regulation of spermatogenesis and retinogenesis^22–26^. Dysregulation of ELAVL2 and its gene expression networks was proposed to contribute to the development of neurological disorders, including autism spectrum disorder^27^. However, the role of ELAVL2 in human cancer development remained elusive. In this study, we provide new insights into the role of ELAVL2 in GBM by investigating the clinical and molecular aspects of ELAVL2 in tumor progression and show that ELAVL2 may act as a potential tumor suppressor by regulating the mRNA stability of downstream effectors, thereby suppressing aggressive MES transformation in GBM.

## Results

### ELAVL2 deletion is associated with glioma progression

**Fig. 1:**
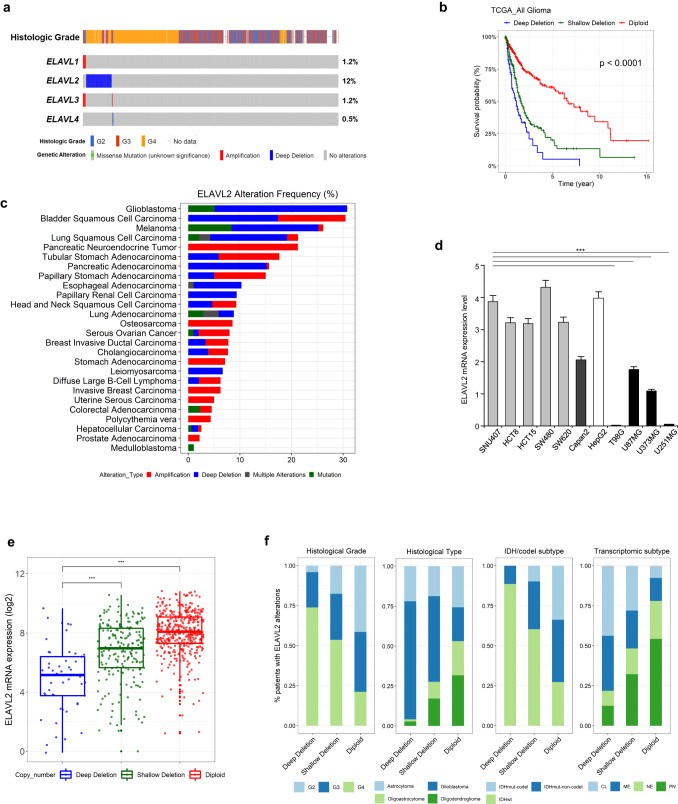
Copy number alteration status of ELAVL2 in glioma patients. **a** The copy number alteration status of ELAVL family members (ELAVL1-4) in grade II, III, and IV gliomas was obtained from the Merged Cohort of LGG and GBM (TCGA, Cell 2016) using cBioPortal platform. **b** Kaplan–Meier survival analysis of glioma patients based on their ELAVL2 copy number alteration status. *p*, log-rank test. **c** Comparison of ELAVL2 copy number alteration frequency across multiple cancer databases, which were accessed through the Pan-cancer analysis of whole genomes (ICGC/TCGA, Nature 2020) in cBioPortal platform. **d** qRT-PCR analysis was performed to measure basal mRNA expression levels of ELAVL2 in various cancer cell lines: colorectal adenocarcinoma (light gray); pancreatic adenocarcinoma (dark gray); hepatocellular carcinoma (white); and glioblastoma (GBM) (black). Data are presented as the mean ± SD (*n* = 3). ***p < 0.001 between each GBMs (T98G, U87MG, U373MG, U251MG) and other cancer cell lines. **e** Boxplot comparing ELAVL2 mRNA expression levels and copy number alteration status. ***p < 0.001. **f** Hundred percent stacked bar charts showing the associations between ELAVL2 deletion status and clinical and molecular parameters of glioma. IDHmut, IDH-mutant; codel, co-deletion; IDHwt, IDH-wildtype; CL, classical; ME, mesenchymal; NE, neural; PN, proneural. Datasets used in **a** and **f** were obtained from Merged Cohort of LGG and GBM in cBioPortal platform, and those used in **e** were from TCGA_GBMLGG dataset in GlioVis online platform. Details are provided in Materials and Methods.

The genomic alteration status of the ELAVL family (ELAVL1-4) was first assessed using the copy number alteration (CNA) data from the Cancer Genome Atlas (TCGA) database. Strikingly, only ELAVL2 was found to be aberrantly deleted at high frequency in glioma patients (12%) (Fig. 1a). When cancer type was restricted to only GBM samples, ELAVL2 was also found to be deleted the most (19%) compared to other ELAVL members (Supplementary Fig. 1a). Notably, overall survival of glioma patients was found to be affected by the ELAVL2 deletion status (Fig. 1b). We then sought to determine if ELAVL2 deletion specifically occurs in GBM compared to other types of cancer. The alteration frequency of ELAVL2 in various cancer datasets was ranked, and the result showed that ELAVL2 was deleted at the highest level in GBM compared to other cancers, suggesting that ELAVL2 deletion is one of the frequent genomic alterations in GBM (Fig. 1c). To confirm the low expression level of ELAVL2 in GBM cell lines, we assessed the mRNA expression levels of ELAVL2 in various cancer cell lines, including GBM (T98G, U87MG, U373MG, and U251MG), hepatocellular carcinoma (HepG2), pancreatic cancer (Capan2), and colorectal adenocarcinoma (SNU407, HCT8, HCT15, SW480, and SW620). The result showed that the transcript level of ELAVL2 in GBM cell lines is reduced compared to that of other cancer cell lines (Fig. 1d). Similarly, the mRNA expression level of ELAVL2 was found to be significantly affected by its CNA status (Fig. 1e). Furthermore, ELAVL2 was found to be deleted mostly in grade IV glioma, GBM, IDH-wt glioma, and GBMs of classical and MES molecular subtypes, all of which define the aggressive phenotypes of glioma (Fig. 1f). Also, ELAVL2 was expressed at relatively low levels compared to other ELAVL family members in commercial GBM cell lines (Supplementary Fig. 1b). Collectively, these data suggest that ELAVL2 depletion may represent a characteristic feature of GBM, with potential relevance to glioma progression.

### Associations of ELAVL2 mRNA level with clinical and molecular characteristics of glioma

**Fig. 2:**
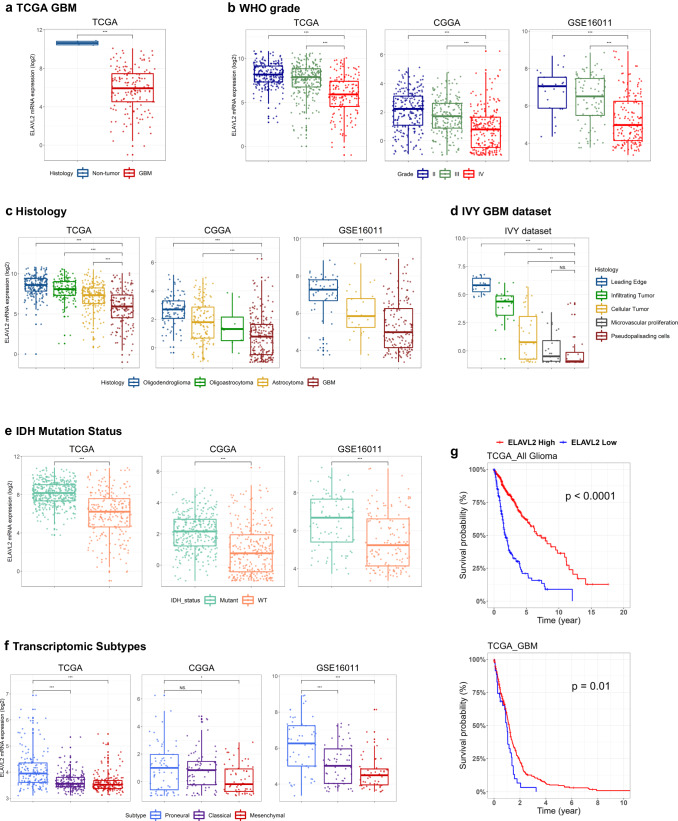
The landscape of clinical and molecular features associated with ELAVL2 mRNA expression level. **a**–**f** Box plots comparing ELAVL2 mRNA expression levels between non-tumor and GBM samples **a**, among different WHO grades **b**, among different histological classifications **c**, among different anatomical regions of GBM **d**, between IDH mutant versus wildtype samples **e** and among different GBM transcriptomic gene signatures **f**. NS non-significant. *p < 0.05, **p < 0.01, ***p < 0.001. **g** Kaplan–Meier survival curve of glioma patients (upper panel) and GBM patients (lower panel) divided based on their ELAVL2 mRNA expression levels. *p*, log-rank test. TCGA GBM U133 microarray dataset was used for **a,** **f** and lower panel of **g**, whereas TCGA_GBMLGG dataset was used for the rest of the TCGA analysis. Four different cohorts (TCGA, CGGA, GSE16011 and IVY GBM) were used as indicated.

To elucidate the expression level of ELAVL2 in clinical and molecular features associated with glioma progression, the mRNA level of ELAVL2 was assessed on various histological and molecular criteria by utilizing several publicly available glioma mRNA datasets retrieved from TCGA, CGGA, IVY GBM, and GSE16011. ELAVL2 expression level was found to be dramatically lower in GBM than that in non-tumor tissues (Fig. 2a), supporting the previous observation that ELAVL2 deletion is a characteristic of GBM. Notably, the expression level of ELAVL2 was the lowest in tumors belonging to WHO grade IV compared to lower grade gliomas (Fig. 2b). Similarly, ELAVL2 expression level was low in the higher histopathological malignancies, being the lowest in GBM and highest in oligodendroglioma (Fig. 2c). Furthermore, ELAVL2 expression was lowest in microvascular proliferation and pseudopalisading cells, which were previously reported to contribute to the aggressiveness of GBM (Fig. 2d)^28^. Furthermore, the expression level of ELAVL2 was low in gliomas with wild-type isocitrate dehydrogenase 1 (IDH1) gene (Fig. 2e), which plays a critical role in glioma progression^1^. Among the 3 gene expression-based transcriptomic signatures, PN and MES GBMs were shown to exhibit better and worse prognosis, respectively^8^. Interestingly, we found that ELAVL2 expression was highest in PN GBM compared to other subtypes (Fig. 2f). To further validate the association of ELAVL2 expression with GBM subtypes, we performed GSEA on TCGA GBM mRNA expression dataset using the MES and PN subtype gene sets. Patients were divided into ELAVL2-high and -low groups, and the gene sets corresponding to each molecular subtype were ranked. The PN and MES gene sets were significantly enriched in ELAVL2-high and -low patients, respectively (Supplementary Fig. 1c, d). It has been reported that genes with similar mechanistic roles are often mutated in a mutually exclusive fashion and that neurofibromatosis type 1 (NF1) gene mutation/deletion predominantly occurs within the MES subtype of GBM^5,29^. Thus, we sought to investigate the possibility of mutual exclusivity in genomic abnormalities between ELAVL2 and NF1, considering the close relationship between the MES subtype and ELAVL2. Our analysis revealed a notable mutually exclusive pattern in the genomic alteration status between ELAVL2 and NF1 (Supplementary Fig. 1e), suggesting the potential mechanistic role of ELAVL2 in driving MES transition in GBM. Furthermore, Kaplan–Meier survival analysis indicated that low expression of ELAVL2 is associated with dismal outcome in both glioma (p < 0.0001) and only GBM datasets (p = 0.01) (Fig. 2g). Taken together, these data indicate that the loss of ELAVL2 is associated with the histological and molecular features of glioma and thus may be used as a novel biomarker for the aggressiveness of gliomas.

### ELAVL2-associated transcriptomic landscape in GBM patients

**Fig. 3:**
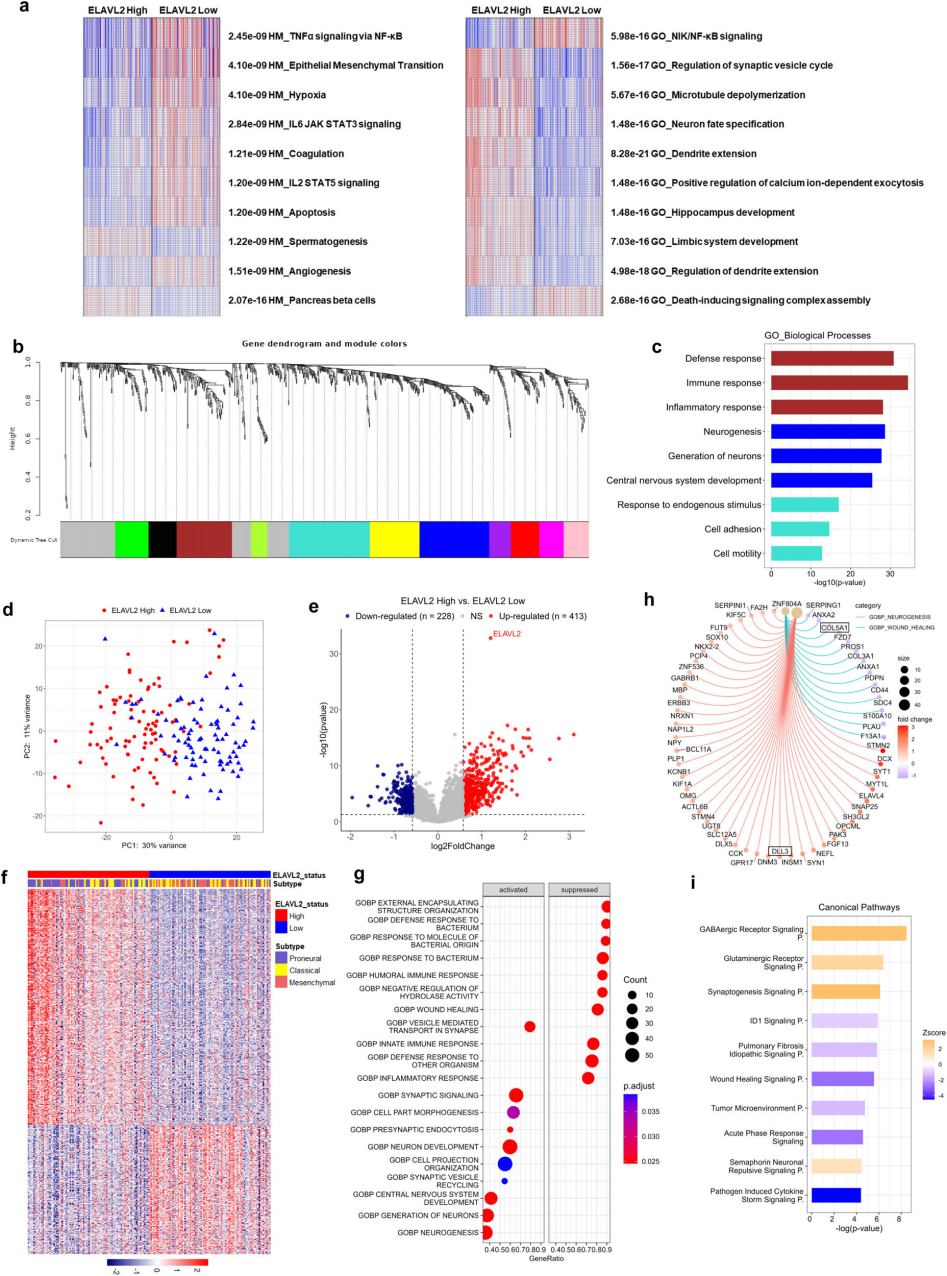
Transcriptomic signatures associated with ELAVL2 mRNA expression levels in GBM. **a** PGSEA enrichment heatmaps of HM (left panel) and GO gene sets (right panel) in the transcriptomic profiles of ELAVL2-high and -low TCGA GBM patients. Red indicates higher expression of gene sets, whereas blue means lower expression. The associated FDR values are indicated next to each gene set. **b** WGCNA was performed on the transcriptomic data of the same set of samples used in **a**. Each color label identifier represents each module, or a cluster of genes with a similar expression pattern across samples. **c** GO analysis on the identified gene sets in turquoise, blue, and brown modules. Bar plot shows the top 3 altered pathways in each module. **d** PCA plot illustrating the transcriptomic difference in the same set of patients used in (**a**). **e** Volcano plot of DEGs in ELAVL2-high vs. ELAVL2-low GBM patients. A number of DEGs were identified as indicated. **f** Heatmap illustrating the expression level of DEGs in ELAVL2-high and -low GBM patients. GBM transcriptomic subtype annotation is shown. **g** Dot plot showing the top 10 enriched GO pathways of biological processes on the DEG profile of ELAVL2-high GBM patients. Activated and suppressed processes are associated with up- and down-regulated genes, respectively. **h** Cnetplot depicting the linkages of genes and biological processes of neurogenesis and wound healing as a network. Two GBM subtype-associated genes, COL5A1 (MES) and DLL3 (PN), are highlighted in the plot. **i** Altered canonical pathways in IPA on the DEG profile of ELAVL2-high GBM patients. Each bar is colored based on the activation z-score of the pathway.

To characterize the transcriptomic signatures associated with ELAVL2 expression level, we first utilized parametric gene set enrichment analysis (PGSEA) using the gene expression profiles of ELAVL2-high and -low TCGA GBM patients (Fig. 3a). The analysis revealed that gene sets indicative of invasiveness of cancer, including TNFα signaling via NF-kB, EMT, and hypoxia, are upregulated in ELAVL2-low GBM patients. On the other hand, gene sets representing normal neuronal processes, such as the regulation of synaptic vesicle cycle and neuron fate specification, are enriched in ELAVL2-high GBM patients. To further dissect the ELAVL2-mediated transcriptomic alterations, we performed weighted gene co-expression network analysis (WGCNA) on ELAVL2-high and -low GBM patients and identified 11 gene modules from a network of 819 genes after applying a soft power value of 5 (scale-free *R*^2^ = 0.8) (Fig. 3b). We then carried out GO analysis on the basis of biological processes using the top three largest modules: turquoise (153 genes), blue (132 genes), and brown (105 genes), and ranked the enriched GO terms. As shown in Fig. 3c, the genes belonging to the turquoise module were found to be associated with cellular movement. Also, the brown and blue modules were enriched in functions related to the inflammatory response and normal neuronal processes, respectively. These network-specific associations suggested distinctive transcriptomic regulations in ELAVL2-high and -low GBM patients.

To gain further insight into the molecular pathways regulated by ELAVL2, we performed differentially expressed gene (DEG) analysis comparing the transcriptomes of ELAVL2-high and -low GBM patients. Principal component analysis (PCA) indicated that ELAVL2-high GBM patients were clearly separated from ELAVL2-low patients, which was further supported by the box plots using PC1 and PC2 values, indicating a significant difference in the transcriptomic profiles along the PC1 dimension. (Fig. 3d, Supplementary Fig. 2). A total of 413 up- and 228 down-regulated DEGs were identified in ELAVL2-high GBM patients when compared with ELAVL2-low patients, which are illustrated in the volcano plot and heatmap (Fig. 3e, f, Supplementary Table 1). Expectedly, the DEG profile of ELAVL2 was associated with the transcriptomic subtype of GBM (Fig. 3f). We then carried out GO analysis on the DEGs and found that pathways related with neuronal functions are activated, whereas those associated with cellular inflammatory response are suppressed in ELAVL2-high GBM patients (Fig. 3g). Gene-concept network analysis revealed that some of the critical GBM subtype-related genes, namely COL5A1 (MES) and DLL3 (PN), are highly associated with ELAVL2-mediated transcriptomic signatures (Fig. 3h). Next, the DEG profile of ELAVL2-high GBM patients was uploaded into IPA and the canonical pathway analysis revealed that EMT-related pathways, including wound healing signaling (z-score: -2.8) and tumor microenvironment pathways (z-score: -1.5), are highly deactivated, suggesting that ELAVL2 may repress MES-associated phenotypes in GBM (Fig. 3i). Overall, these results indicate that ELAVL2 may regulate the transcriptomic profile of GBM with distinct biological consequences and its expression may suppress MES-related pathways.

### ELAVL2 expression inversely correlates with MES signatures in GBM patients

**Fig. 4:**
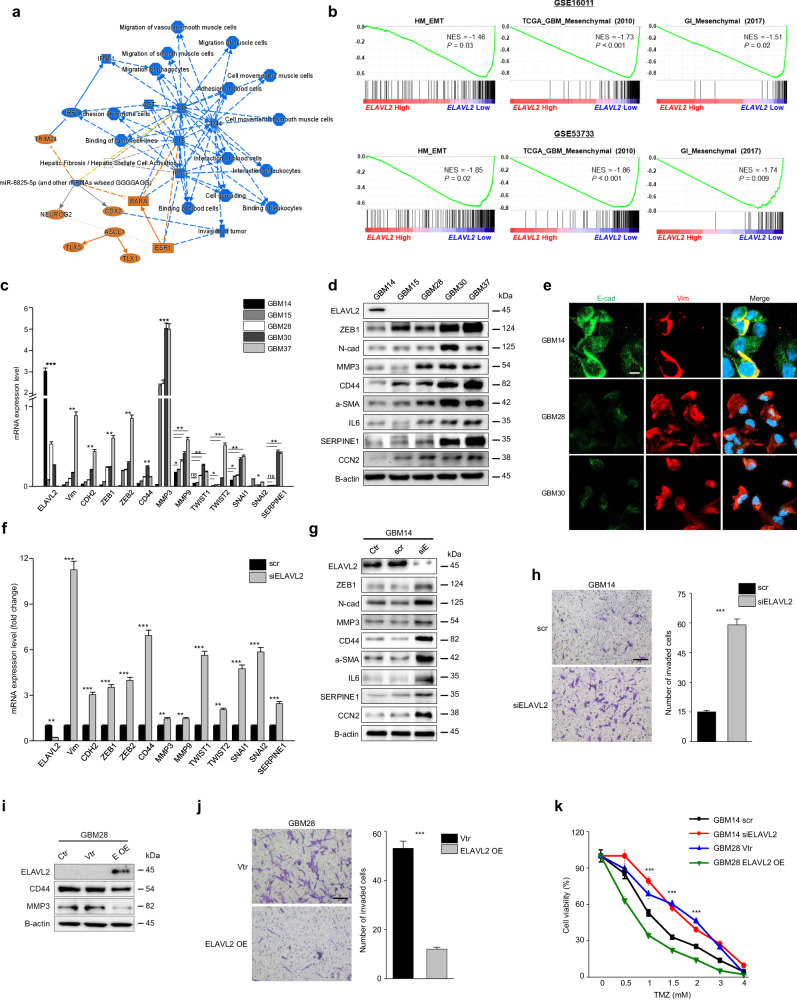
ELAVL2 negatively controls MES molecules and chemoresistance in GBM cell lines. **a** Graphical summary of IPA, which depicts how the major biological themes and molecules are related to one another at the transcriptomic level, was generated on the DEG profile of ELAVL2-high GBM patients. Blue means deactivation, and orange activation. Solid and dotted lines represent direct and indirect interactions, respectively. **b** GSEA results of MES-related gene signatures comparing ELAVL2-high with ELAVL2-low GBM patients in 2 independent GBM cohorts as indicated. Normalized enrichment score (NES), as well as *p*-value, are shown. **c**, **d** RT-qPCR (**c**) and immunoblotting **d** of ELAVL2 and MES-related molecules in patient-derived primary GBM cell lines (GBM14, GBM15, GBM28, GBM30, and GBM37). Data are presented as the mean ± SD (*n* = 3). NS indicates statistically non-significant, *p < 0.05, **p < 0.01, ***p < 0.001 between ELAVL2-high GBM (GBM14) and each of ELAVL2-low GBMs (GBM15 or GBM28 or GBM30 or GBM37). **e** Immunocytochemical for E-cadherin (green), Vimentin (red), and nuclei (blue) were co-stained in GBM14, GBM28, and GBM30. Original magnification x 400. Scale bar, 20 μm. **f**, **g** RT-qPCR (**f**) and immunoblotting (**g**) of ELAVL2 and MES-related molecules in GBM14 transfected with scramble (scr) or siELAVL2 (**f**) and in GBM14 control (Ctr) and transfected with scr or siELAVL2. **g** Data are presented as the mean ± SD (*n* = 3). **p < 0.01, ***p < 0.001 versus the scr group. **h** GBM14 was transfected with scr or siELAVL2 and subjected to invasion (original magnification × 100. Scale bar, 100 μm). Representative images and bar graphs of relative cell invasion (**h**) are shown. All data represent the mean ± SD (*n* = 3). **p < 0.01, ***p < 0.001 versus the scr group. **i**, **j** immunoblotting (**i**) and invasion assay (original magnification × 100. Scale bar, 100 μm) **j** in GBM28 transfected with vector control (vtr) or overexpression vector. Representative images and bar graphs of relative cell invasion **j** are shown. Data are presented as the mean ± SD (*n* = 3). ***p < 0.001 versus vtr group. **k** Cell viability assay of GBM14 transfected with scr or siELAVL2 and GBM28 transfected with vector control or overexpression vector and treated with indicated doses of TMZ for 24 h. Data are presented as the mean ± SD (*n* = 3). ***p < 0.001 versus each scr or vtr group.

In accordance with the previous results, the graphical summary of IPA demonstrated that cancer and inflammatory-associated biological functions, including invasion of tumor and interaction of leukocytes, as well as critical cancer-related genes such as CD44, TNF, and IL-1B, are markedly deactivated in ELAVL2-high GBM patients (Fig. 4a). To further examine the possible involvement of ELAVL2 in the MES transition, we performed GSEA on other public GBM mRNA expression datasets (GSE16011 & GSE53733) obtained from GEO database using 3 distinct MES-related gene sets - HM_EMT, TCGA_GBM_Mesenchymal (2010), and GI_Mesenchymal (2017). Surprisingly, all of these gene signatures were found to be significantly enriched in ELAVL2-low GBM patients in these GBM cohorts (Fig. 4b), enabling us to hypothesize that ELAVL2 might negatively control the expression of MES-related molecules.

To confirm an inverse correlation of ELAVL2 with MES-related molecules in-vitro level, we performed RT-qPCR using representative MES markers. Several patient-derived primary GBM cell lines (GBM14, 15, 28, 30, and 37) and commercial GBM cell lines (T98G, U251MG, U87MG, and U373MG) were used for the analysis. Among the cell lines tested, GBM14, U87MG, and U373MG cell lines expressed higher ELAVL2 levels compared to the other GBM cell lines (Fig. 4c, Supplementary Fig. 3a). As expected, we found that ELAVL2 expression level negatively correlated with the indicated MES genes (Fig. 4c, Supplementary Fig. 3a). Such a negative correlation between the expression pattern of ELAVL2 and MES markers was also verified at protein level by immunoblotting (Fig. 4d, Supplementary Fig. 3b). Next, to determine whether ELAVL2-low GBM cells are indeed close to the MES phenotype, we performed immunocytochemistry of the EMT markers – E-cadherin (green) and Vimentin (red) – on GBM14, GBM28, and GBM30. As shown in Fig. 4e, GBM14 (ELAVL2-high) was characterized by increased fluorescence intensity of E-cadherin and decreased intensity of Vimentin, whereas GBM28 and GBM30 (ELAVL2-low) displayed an opposite expression pattern. Taken together, these results show that the expression of ELAVL2 is negatively correlated with that of EMT-related molecules at both mRNA and protein levels, suggesting that the loss of ELAVL2 may promote glioma progression by facilitating MES transition.

### ELAVL2 regulates expression of MES molecules and aggressiveness in GBM cell lines

To examine whether ELAVL2 negatively controls MES-related molecules and cellular phenotypes, we designed 3 ELAVL2-targeting siRNAs to perform loss-of-function studies. siRNA #1 was found to most effectively decrease ELAVL2 protein level and thus used for the rest of the analyses involving ELAVL2 knockdown in ELAVL2-high GBM cell lines (Supplementary Fig. 4a). Surprisingly, the expression of all MES markers tested profoundly increased after ELAVL2 knockdown at both mRNA and protein levels in GBM cells (Fig. 4f, g, Supplementary Fig. 4b, c). These results suggest that ELAVL2 may exert its tumor suppressive effect in GBM by negatively regulating the critical MES-related molecules. To determine the functional outcome of ELAVL2 knockdown, we assessed changes in invasion, migration, and proliferation in GBM cell lines. As shown in Fig. 4h, Supplementary Fig. 4d–f and 5a, b, all these capacities were increased upon ELAVL2 knockdown compared to controls. Furthermore, we transfected the ELAVL2 overexpression vector into the ELAVL2-low GBM28 cell line to examine the reversal effects in contrast to ELAVL2 knockdown cells. Notably, MES marker protein levels and the number of invaded cells decreased in ELAVL2-overexpressed GBM cells (Fig. 4i, j). Additionally, we investigated whether the function of ELAVL2 remained unchanged or was altered after different durations of siELAVL2 transfection in GBM14. While we did not observe a complete restoration of ELAVL2 protein expression 3 weeks after siRNA transfection, we noticed a decrease in invasion capacity, along with changes in the expression of MES-related proteins in GBM14 cells that were transfected with siELAVL2 for 3 weeks (Supplementary Fig. 6a, b). These findings suggest that MES phenotypes are indeed reversible, highlighting the plastic nature of GBM cells.

Next, we investigated whether silencing or overexpressing ELAVL2 affected the sensitivity of GBM cells to TMZ, as the MES phenotype is closely linked to chemoresistance. Our results demonstrated that ELAVL2-depleted GBM cells exhibited higher viability compared to ELAVL2-overexpressed GBM cells when treated with increasing doses of TMZ for 24 h, indicating that ELAVL2 may regulate chemoresistance in GBM cells (Fig. 4k, Supplementary Fig. 7b). Furthermore, we used SYTOX green staining to visualize and compare cell death in siELAVL2- and scr-treated GBM cells after 2 mM TMZ treatment. Consistent with the cell viability result, siELAVL2-transfected cells exhibited significantly less cell death compared to controls (Supplementary Fig. 7a, c). Collectively, these data indicate that loss of ELAVL2 promotes MES-related phenotypes in GBM cells and also induces TMZ resistance partly by up-regulating EMT-related molecules.

### High ELAVL2 protein level is associated with a favorable survival of glioma patients

**Fig. 5:**
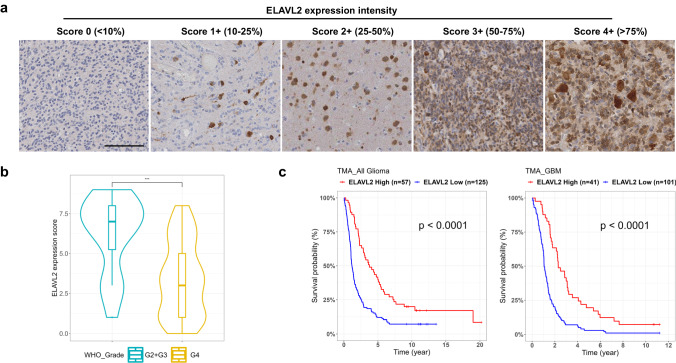
ELAVL2 protein levels are reduced in aggressive GBM and inversely correlate with patient survival. **a** Representative images of IHC staining on TMAs based on the intensity scores of ELAVL2. Scale bar, 100 μm. **b** Violin plot showing the distribution of ELAVL2 expression scores between grade II, III and grade IV gliomas. ***p < 0.001. **c** Kaplan–Meier survival curves of all glioma (left panel) and GBM (right panel) patients divided based on the intensity score of ELAVL2 IHC staining. *p*, log-rank test.

To further substantiate the prognostic role of ELAVL2 in glioma, we analyzed the protein expression level of ELAVL2 in 182 glioma patients with follow-up information across four TMA sets (TMA1573, 2248, 2249, 2758) using an anti-ELAVL2 antibody. Detailed clinical information of the glioma patients is provided in Supplementary Table 2. Patients were divided into ELAVL2-high and -low groups based on IHC staining intensity score. Representative images of ELAVL2 staining with different intensity scores are shown in Fig. 5a. ELAVL2 intensity scores were higher in grade II/III gliomas than in grade IV (Fig. 5b). Importantly, glioma patients with high ELAVL2 protein expression levels are significantly associated with better survival than those with low expression, and the result remained significant when only GBM patients were analyzed (Fig. 5c). These results indicate that ELAVL2 protein expression level is associated with tumor progression and also favorable survival rate of glioma patients, and thus, may be used as a prognostic and diagnostic biomarker for gliomas.

### ELAVL2 modulates the expression of SH3GL3 and DNM3 as downstream effectors

**Fig. 6:**
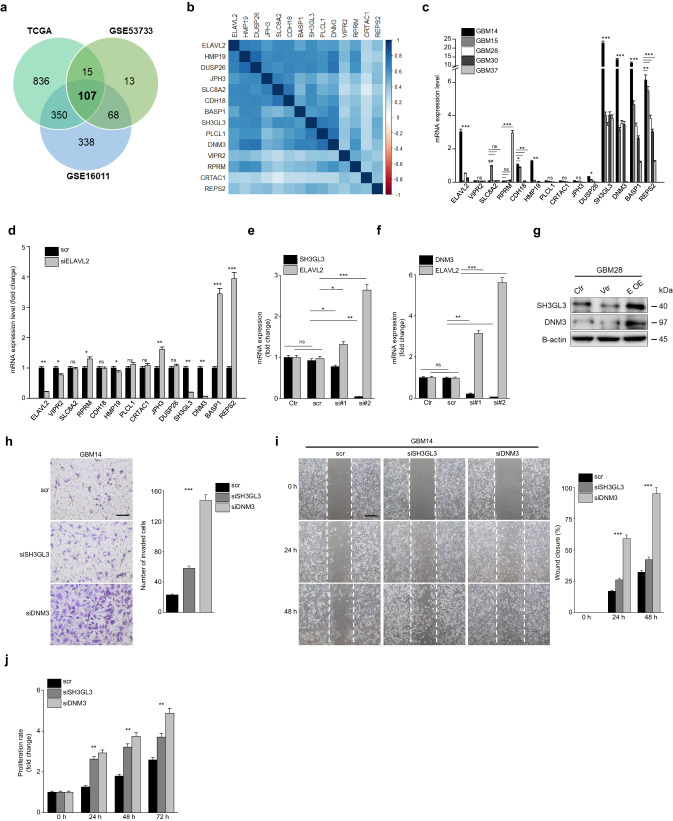
ELAVL2 regulates SH3GL3 and DNM3 to suppress MES-associated phenotypes. **a** Venn diagram displaying a number of overlapping genes that are positively correlated with ELAVL2 mRNA expression in 3 different GBM cohorts. **b** Gene correlation plot of ELAVL2 and 13 candidate target genes in TCGA GBM patients. **c** Basal mRNA expression levels of ELAVL2 and 13 candidate target genes in primary GBM cell lines (GBM14, GBM15, GBM28, GBM30, and GBM37). Data are presented as the mean ± SD (*n* = 3). NS indicates statistically non-significant, *p < 0.05, **p < 0.01, ***p < 0.001 between ELAVL2-high GBM (GBM14) and each of ELAVL2-low GBMs (GBM15 or GBM28 or GBM30 or GBM37). **d** mRNA expression levels of the 13 candidate target genes in GBM14 after ELAVL2 knockdown. All data represent the mean ± SD (*n* = 3). NS indicates statistically non-significant, *p < 0.05, **p < 0.01, ***p < 0.001 versus the scr group. **e**, **f** mRNA expression levels of ELAVL2 and SH3GL3 (**e**) and ELAVL2 and DNM3 (**f**) after SH3GL3 (**e**) or DNM3 (**f**) knockdown in GBM14. All siRNA-transfected cells were normalized to GBM14 control (Ctr). All data represent the mean ± SD (*n* = 3). NS indicates statistically non-significant. *p < 0.05, **p < 0.01, ***p < 0.001 versus the corresponding scr group. **g** Immunoblotting of SH3GL3 and DNM3 in basal GBM28 and GBM28 transfected with vector control or overexpression vector. **h**–**j** Invasion (original magnification × 100. Scale bar, 100 μm) (**h**) wound healing (original magnification × 40. Scale bar, 250 μm) (**i**) and proliferation assays (**j**) in GBM14 transfected with scr or siSH3GL3 or siDNM3. Representative **i**mages and bar graphs of relative cell invasion (**h**) and wound closure (**i**) are displayed. All data represent the mean ± SD (*n* = 3). **p < 0.01, ***p < 0.001 versus the scr group.

To identify potential downstream regulatory factors responsible for mediating the tumor-suppressive effect of ELAVL2, we conducted gene correlation analyses across three independent datasets (TCGA, GSE16011, and GSE53733) using a stringent cutoff of correlation *p*-value ≤ 10^-4^. We identified 107 genes whose expressions were significantly and positively correlated with ELAVL2 across all three datasets (Fig. 6a and Supplementary Table 3). We then conducted a thorough literature search to identify genes with known roles in inhibiting EMT or acting as potential tumor suppressors in glioma or other cancers. As a result, we selected 13 candidate target genes whose mRNA expression levels were strongly correlated with ELAVL2 (Fig. 6b), thereby implicating these genes as potential downstream effectors of ELAVL2-mediated tumor suppression.

To further validate such a positive correlation between ELAVL2 and potential target molecules at in vitro level, we performed RT-qPCR and showed that ELAVL2-high GBM cells (GBM14, U87MG, and U373MG) expressed CDH18, HMP19, DUSP26, SH3GL3, DNM3, BASP1, and REPS2 at significantly higher levels compared to ELAVL2-low GBM cells (Fig. 6c, Supplementary Fig. 8a). Next, we carried out a loss-of-function experiment using siELAVL2 in GBM14 and U373MG. Interestingly, SH3GL3 and DNM3 were the most significantly down-regulated molecules after ELAVL2 knockdown suggesting that these 2 molecules may be downstream effectors of ELAVL2 (Fig. 6d, Supplementary Fig. 8b). Furthermore, knockdown of either SH3GL3 or DNM3 resulted in a significant increase in ELAVL2 mRNA levels in GBM14, suggesting that ELAVL2 expression may compensate for the loss of these molecules (Fig. 6e, f). Importantly, SH3GL3 and DNM3 protein levels were increased when ELAVL2 was overexpressed in GBM28 (Fig. 6g).

Notably, GSEA analysis revealed that PN transcriptomic signatures are enriched in GBM patients with high SH3GL3 or DNM3 expression levels. Although not statistically significant except two, a clear trend was observed that the MES-related gene signatures (MES subtype and EMT gene sets) are enriched in patients with low SH3GL3 or DNM3 expression levels (Supplementary Fig. 8c, d). These results propose the potential signaling axis of ELAVL2-SH3GL3/DNM3 in regulating aggressive MES transition in GBM, conferring favorable overall survival in SH3GL3-high and DNM3-high GBM patients (Supplementary Fig. 8e).

### ELAVL2 regulates mRNA stability of SH3GL3 and DNM3 in an m6A-dependent fashion

To investigate the impact of SH3GL3 and DNM3 knockdown at cellular level, we carried out invasion, proliferation, and cell migration assays using siSH3GL3 or siDNM3. As shown in Fig. 6h–j and Supplementary Fig. 9a–c, downregulation of SH3GL3 or DNM3 significantly increased the invasion, migration, and proliferation abilities of GBM14 and U373MG cells. Furthermore, we upregulated SH3GL3 and DNM3 using N-acetyl cysteine (NAC), an ROS scavenger, and found that both the protein and mRNA levels of MES-related molecules decreased after NAC treatment, suggesting the EMT-inhibitory effects of these downstream effectors of ELAVL2 (Supplementary Fig. 10a, b).

**Fig. 7:**
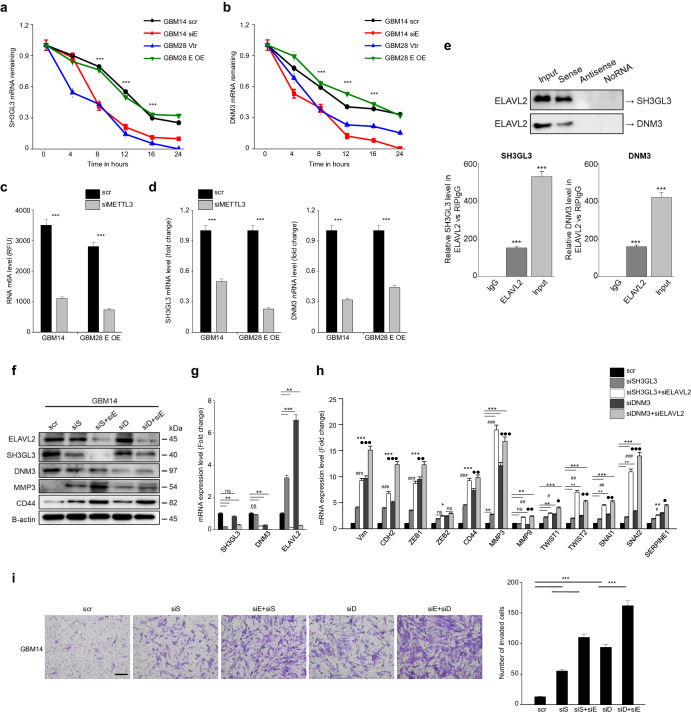
ELAVL2 regulates MES phenotypes in GBM by modulating the mRNA stability of SH3GL3 and DNM3, potentially through an m6A-dependent mechanism. **a**, **b** Changes in mRNA stability of SH3GL3 (**a**) and DNM3 (**b**) were observed in GBM14 transfected with scr or siELAVL2 and GBM28 transfected with vector control or overexpression vector. All data represent the mean ± SD (*n* = 3). ***p < 0.001 versus each scr or vtr group. **c**, **d** m6A methylation (**c**) and mRNA level of SH3GL3 and DNM3 (**d**) were quantified in GBM14 transfected with scr or siMETTL3 and GBM28 ELAVL2 overexpressed cells transfected with scr or siMETTL3. All data represent the mean ± SD (*n* = 3). ***p < 0.001 versus to scr group. **e** Immunoblotting of ELAVL2 which was pulled down by a SH3GL3 or DNM3 sense RNA probe, but not by an antisense RNA probe or no RNA (top panel). RIP assays with qRT-PCR were conducted on RNA pulled down by an ELAVL2 antibody in ELAVL2 overexpressed GBM28 cells (bottom panel). Data are presented as the mean ± SD (*n* = 3). ***p < 0.001 versus the IgG group. **f**–**i** Protein (**f**) mRNA levels of SH3GL3, DNM3, ELAVL2 (**g**) MES markers (**h**) and invasion assay (original magnification × 100. Scale bar, 100 μm) (**i**) in GBM14 transfected with the following conditions: scr, siSH3GL3, siSH3GL3 + siELAVL2, siDNM3, and siDNM3 + siELAVL2. All cells were normalized to scr. Data are presented as the mean ± SD (*n* = 3). NS indicates statistically non-significant, *p < 0.05, **p < 0.01, ***p < 0.001 versus the scr group. ^#^*p* < 0.05, ^##^*p* < 0.01, ^###^*p* < 0.001 between siSH3GL3 and siSH3GL3 + siELAVL2 group. ^●^*p* < 0.05, ^●●^*p* < 0.01, ^●●●^*p* < 0.001 between siDNM3 and siDNM3 + siELAVL2 group.

To examine the post-transcriptional mechanism by which ELAVL2 regulates SH3GL3 and DNM3, we first examined the mRNA half-life of SH3GL3 and DNM3 in both ELAVL2-depleted and ELAVL2-high GBM cells. Our findings demonstrated that ELAVL2 depletion led to a decrease in the stability of SH3GL3 and DNM3 mRNA transcripts, while ELAVL2 overexpression had the opposite effect, suggesting that ELAVL2 may play a pivotal role in stabilizing these downstream molecules (Fig. 7a, b). Subsequently, to explore the potential link between ELAVL2 and m6A modification, we employed siRNA to knockdown METTL3, an m6A writer, in ELAVL2-high GBM cells and observed the reduced mRNA expression levels of SH3GL3 and DNM3, suggesting the potential relevance of m6A modification in ELAVL2 function (Fig. 7c, d). Based on this observation, we then sought to determine whether ELAVL2 protein directly binds to SH3GL3 and/or DNM3 mRNA transcripts. Our RNA pull-down assay demonstrated direct binding of the ELAVL2 protein to the sense sequences of SH3GL3 and DNM3 transcripts in ELAVL2-high GBM cells (Fig. 7e; top panel). Moreover, an RNA immunoprecipitation (RIP) assay using an ELAVL2 antibody confirmed the binding interaction between endogenous ELAVL2 and the SH3GL3 or DNM3 transcripts in ELAVL2-high GBM cells (Fig. 7e; bottom panel). A proposed mechanism of ELAVL2-mediated repression of MES-related phenotypes via SH3GL3 and DNM3 in GBM is illustrated in Supplementary Fig. 11. Taken together, our data suggest that ELAVL2 protein may directly bind to downstream mRNAs and modulate the stability of SH3GL3 and DNM3, potentially in an m6A-dependent manner.

### ELAVL2-SH3GL3/DNM3 axis regulates MES phenotypes in GBM cell lines

Next, we examined protein and mRNA levels of ELAVL2, SH3GL3, DNM3, and MES markers in GBM14 transfected with following conditions: scr; siSH3GL3; siSH3GL3 + siELAVL2; siDNM3; and siDNM3 + siELAVL2. Interestingly, co-treatment of siSH3GL3 + siELAVL2 or siDNM3 + siELAVL2 significantly reduced the expression levels of SH3GL3 and DNM3, respectively, at higher degrees than a single siSH3GL3 or siDNM3 treatment (Fig. 7f, g). Also, while siSH3GL3 or siDNM3 treatment greatly increased ELAVL2 protein and mRNA expression, co-treatment with siELAVL2 reduced its expression, suggesting that both SH3GL3 and DNM3 may indeed be downstream regulators of ELAVL2 as shown before (Fig. 7f, g). Similarly, while a single treatment of each siRNA was enough to significantly increase the expression levels of several MES markers, including Vim, CDH2, ZEB1, CD44, MMP3, TWIST1, SNAI1, SNAI2, and SERPINE1, co-treatment with siELAVL2 increased their expression even at higher levels compared to both scramble controls and single siRNA-treated samples (Fig. 7h). All these results could be reproduced in U373MG (Supplementary Fig. 9d, e). Importantly, we also showed that co-treatment of siELAVL2 and siRNA for each of the downstream molecules resulted in a higher invasive capacity compared to a single siRNA treatment (Fig. 7i). Of note, knockdown of DNM3 resulted in a greater increase in MES traits in GBM cells than that of SH3GL3, which suggests that DNM3 might play a more critical role than SH3GL3 in suppressing the MES features of GBM. Collectively, these results imply the existence of ELAVL2-SH3GL3/DNM3 signaling axis in preventing the aggressive MES characteristics of GBM and, thus, modulation of these molecules may be a novel and effective therapeutic strategy for GBM patients.

## Discussion

Extensive research has been conducted to decipher the genetics of GBM at multi-omics scales and revealed that the heterogeneous nature of the tumor is plastic rather than fixed. Such plastic nature of the genetics of GBM is well described through the existence of multiple transcriptomic signatures at intratumoral level and the cellular transitions among them^6,30^. Transition from one transcriptomic subtype to another has been considered as one of the major mechanisms by which GBM cells overcome the therapeutic stresses and survive. Especially, PMT was initially noted as one of the most frequent phenomena observed in recurrent high-grade gliomas after extensive therapies^8,31^. Although it was later noted that PMT is not as frequent as it is expected to be, MES transition of tumor cells poses a significant challenge for the development of effective therapies for GBM^1,4,7,8^. In the present study, we showed that ELAVL2, which is the most deleted ELAVL family member in GBM, is associated with MES transcriptomic signature and may control MES transformation through the SH3GL3/DNM3-mediated downstream signaling axis. Such a scenario is schematically illustrated in Supplementary Fig. 11.

Because of an intimate relationship between RBPs and post-transcriptional gene modification, an increasing number of studies have placed RBPs as one of the most important layers related to tumorigenesis and tumor progression in various cancers^17^. Among the ELAVL members, ELAVL1 is a relatively well-studied RBP in the context of cancer^32^. ELAVL1, which is located on chr19p13.2, was reported to play critical roles in various tumors, including glioma, mainly by stabilizing target mRNAs upon binding^33–35^. Filippova et al. showed that ELAVL1 is highly expressed in primary GBMs and its overexpression led to the development of chemoresistance, whereas its silencing suppressed tumor growth by promoting apoptosis^35^. A list of known ELAVL1 targets in GBM is reviewed by Guha et al. ^36^. Based on this, it is plausible that ELAVL1 and ELAVL2, although they belong to the same family of RBP, may act in opposing manners in regulating GBM development and progression, raising the possibility that the balance between the two may be critical in GBM. Further research is needed to understand their potential reciprocal roles in GBM.

The primary function of ELAVL2 has mainly been associated with neuronal differentiation, development and function. Together with ELAVL3, ELAVL2 was initially reported to be required for inducing neuronal phenotype in both mammalian central and peripheral nervous systems^22^. Berto et al. found that ELAVL2-regulated co-expression networks are enriched for neurodevelopmental and synaptic genes important for normal brain function, and their dysregulation might be associated with the development of autism spectrum disorder^27^. Additionally, the post-transcriptional network underlying the formation of primordial follicles in the mouse ovary was found to be largely affected by Elavl2, highlighting a functional diversity of ELAVL2^24^. Of note, Elavl2 was found to promote the translation of Ddx6, whose deletion led to misregulated PI3K-AKT signaling and subsequent premature oocyte enlargement. This result implies that ELAVL2 may also regulate PI3K-AKT signaling, which was reported to induce EMT and enhance tumor aggressiveness^37^. All these studies linking ELAVL2 and its obligatory function in neuronal cells point out that ELAVL2 favors the pro-neuronal state of GBM cells, while its loss may promote the transition towards the MES state.

Interestingly, in contrast to the tumor suppressive role of ELAVL2 observed in our study, tumor promoting effects of ELAVL2 have also been reported. The ELAVL2-CDKN1A axis was found to contribute to the development of paclitaxel resistance in esophageal squamous cell carcinoma by inhibiting cell apoptosis^38^. Furthermore, it was recently reported that ELAVL2 and ELAVL4 activated glycolysis pathway under glucose deprivation condition and their high expression levels were responsible for the development of chemoresistance to paclitaxel in ovarian cancer cells^39^. Such contradictory roles of ELAVL2 underpin its functional plasticity that makes ELAVL2 a “Swiss-Knife” gene whose function is selectively regulated in a tissue- and cancer-specific manner.

ELAVL2 is located on chr9p21, and along with chr10 loss, chr9p deletion has been considered one of the most frequent genomic alterations in GBM, implying the presence of potential tumor suppressors on these deleted chromosomal segments^40,41^. Crespo et al. reported that the chromosome 9p21.3 from the 21,978,443 bp to the 22,119,128 bp position was the most frequently deleted homozygous segments in GBM, which included CDKN2A (52%), CDKN2B and CDKN2BAS (48%), MTAP (26%), ELAVL2 (11%), and TUSC1 (11%)^42^. They initially proposed MTAP as a potential tumor suppressor, because it was the only homozygously deleted gene, and its copy number was significantly correlated with mRNA expression. However, subsequent research found that the loss of MTAP is not associated with GBM aggressiveness, suggesting that certain genes on chr9p, rather than the whole segmental loss, may be individually associated with tumor progression in GBM^43^.

In this study, a combination of *in-silico* and in-vitro analyses revealed that SH3GL3 and DNM3 are putative downstream molecules of ELAVL2. Their expression levels were largely affected by ELAVL2, and their downregulation led to increased invasion, proliferation, and migration of GBM cell lines, accompanied by up-regulation of critical MES effectors. Tumor suppressive activity of SH3GL3 in GBM was recently reported in another study^44^. Overexpression of SH3GL3 decreased neoplastic potentials of GBM cells by inhibiting STAT3 nuclear localization. STAT3 is well-known to be closely related with EMT in driving tumor aggressiveness, and was also identified as one of the master transcriptional regulators for MES GBM^8,45^. Of note, STAT3 was identified as one of the most deactivated upstream regulators in the transcriptomic profile of ELAVL2-high GBM patients in IPA (data not shown). Thus, it is plausible that ELAVL2 hinders the MES process partly through SH3GL3-mediated repression of STAT3. Nonetheless, a tumor promoting function of SH3GL3 was reported in myeloma, suggesting a cancer-specific regulatory activity of ELAVL2-associated downstream molecules^46^. In terms of DNM3, its tumor suppressive function has been reported in various malignancies, including cervical and lung cancers^47,48^. However, its role in GBM progression seems controversial. DNM3 was found to be a direct target of miR-221, which was shown to have tumor promoting function in glioma^49^. On the other hand, a high expression of DNM3 was detected in recurrent GBM, which had stronger growth capacity and lethality than primary GBM in the mouse model^50^. Noteworthy, it was shown in our study that the MES inhibitory effect of DNM3 was greater than that of SH3GL3, implying that ELAVL2 represses the EMT-associated phenotypes primarily through DNM3-mediated repression of MES molecules. Of note, mRNA expression levels of BASP1 and REPS2, which were also positively correlated with that of ELAVL2 in GBM cells, were found to be strongly upregulated after ELAVL2 knockdown (Fig. 6c, d, Supplementary Fig. 8a, b). This result implies that BASP1 and REPS2 may function as upstream regulators of ELAVL2. Since both BASP1 and REPS2 have been shown to possess tumor-suppressive activities^51,52^, it will be worthy of further investigation to decipher their potential signaling network with ELAVL2.

The functions of m6A writers, readers, and erasers suggest that m6A modifications play a role in GBM tumor maintenance^53^. However, the role of one m6A writer, METTL3, in GBM is still controversial, highlighting the complexity of m6A RNA modifications in this context^54,55^. Given that ELAVL2 and other Hu family members like ELAVL1 have been reported to stabilize matrix metalloproteinase 9 (MMP9) mRNA in neurons^56^, we hypothesized that ELAVL2 may function as an RNA stabilizer through interaction with target m6A RNA. Accordingly, our RNA-protein interaction experiments, combined with reduced m6A levels and downstream mRNA expression upon METTL3 knockdown, collectively support the hypothesis that ELAVL2 may directly bind to and modulate downstream mRNA stability, potentially in an m6A-dependent manner. Furthermore, we conducted experiments with various drugs to induce SH3GL3 and DNM3 expression. Interestingly, we found that these molecules increased in GBM cells treated with NAC, a ROS scavenger, suggesting that the SH3GL3/DNM3-EMT axis may be regulated by additional unknown factors that may function in ROS-dependent pathways (Supplementary Fig. 10a, b).

In conclusion, we identified ELAVL2 as a potential tumor suppressor in GBM by regulating the mRNA stability of EMT-inhibitory molecules. The CNA status and mRNA expression level of ELAVL2 were found to correlate with clinical and molecular features of glioma, and its loss was associated with aggressive MES transition and unfavorable survival rates of GBM patients at all genomic, mRNA, and protein levels. Also, our findings regarding the deletion status of ELAVL2 and the subsequent alteration in mRNA expression levels may present a novel molecular pathway in glioma development and progression. Our findings may open a new avenue toward the development of new therapeutic rationales designed to reverse therapy-resistant cell states of GBM.

## Methods

### Cell lines and cell culture

Patient-derived primary GBM cell lines (GBM14, 15, 28, 30, and 37) and commercial GBM cell lines (U87MG, T98G (ATCC), U251MG, and U373MG (Sigma-Aldrich)) were cultured in Dulbecco’s modified Eagle’s high glucose medium (SH30243.01, Hyclone) supplemented with 10% heat-inactivated fetal bovine serum (FBS) (2357664RP, Gibco) and 1% antibiotic-antimycotic (15240062, Gibco). Additionally, SNU407, HCT8, HCT15, SW480, SW620, Capan2, and HepG2 were purchased from Korean cell line bank and cultured according to the provider’s instructions. All cells were maintained at 37 °C in a 5% CO_2_ incubator and tested regularly for mycoplasma contamination by Mycoplasma Detection Kit (rep-mys-20, InvivoGen). In compliance with the Declaration of Helsinki, this study was approved by the Institutional Review Board of Seoul National University Hospital (IRB No. H-0507-509-153), and all subjects provided written informed consents.

### Cell migration, invasion, and proliferation assays

To measure the cell migration ability, cells were plated in 6-well plates and a straight line was scraped with a 200 μl pipet tip when cells reached 80% confluence. After washing with 1 x phosphate-buffered saline (PBS), cells were exposed to serum-free media and wound closure was measured at 24 h and 48 h time points using ImageJ (National Institutes of Health)^57^. For invasion assay, 24-well transwell chambers with a pore size of 8 μm (CLS3470-46EA, Corning) were used after pre-coating the membrane with Matrigel (12.5%) (354230, Corning). 5 × 10^3^ cells/well were placed in the upper chamber in serum-free media, while the lower chamber was filled with media supplemented with 10% FBS. After 48 h, cells were fixed in paraformaldehyde (4%) and stained with 5% crystal violet solution (548-62-9, Sigma-Aldrich). Invaded cells in the lower chamber were quantified by counting crystal violet-stained cells in 4 randomly selected fields under an optical microscope. For cell proliferation assay, 1 × 10^3^ cells/well were seeded in 96-well plates and incubated with MTS solution (G358, Promega) at 24 h, 48 h, and 72 h time points. Absorbance was measured at 490 nm after 1 h of incubation using Varioskan Lux (Thermo).

### Cell viability and death assays

For the CCK-8 assay, 1 × 10^3^ cells/well were seeded in 96-well plates and incubated for 24 h prior to TMZ (85622-93-1, Sigma-Aldrich) treatment. Different concentrations of TMZ were added and incubated for 24 h. Then, 10 μl of CCK-8 solution (CK04-13, Dojindo) was added to each well and incubated for an additional 1 h. The absorbance was measured at 450 nm using Varioskan Lux (Thermo) and background absorbance of the medium was subtracted. For the cell death assay, cells were treated with DMSO or 2 mM TMZ for 24 h. SYTOX™ Green (10768273, Invitrogen) nucleic acid stain was then added, and the fluorescence was measured under a fluorescence microscope (Leica DMI8).

### RNA extraction and reverse transcription-quantitative polymerase chain reaction

Total RNA was isolated from cells using RNeasy Mini Kit (74106, QIAGEN) according to the manufacturer’s instructions, and 1 μg of RNA was used as a template to synthesize cDNA using AccuPower® CycleScript RT PreMix (K-2044, Bioneer). Quantitative real-time PCR analysis was performed on QuantStudio 3 real-time PCR system (Applied Biosystems) using AccuPower® 2X GreenStar™ qPCR Master Mix (2203E, Bioneer). All values were normalized relative to GAPDH mRNA level and the relative target mRNA levels were determined using mathematical expression 2^− (ΔΔCt)^. The sequences of PCR primers are provided in Supplementary Table 4.

### RNA interference and gene transfection

To generate cells that stably overexpress ELAVL2, pUCIDT(Amp)-ELAVL2 (Integrated DNA Technologies) was digested by EcoR I and xba I, and ELAVL2 (NM_004432.5) fragment was inserted into empty pcDNA3.1 (Invitrogen) vector. The sequences of the resulting plasmids ELAVL2 were verified by direct sequencing. Small-interfering RNAs (siRNAs) were purchased from Bioneer, Korea. The sequences of siRNAs used in this study are provided in Supplementary Table 5. Cells at 60-70% confluence were transfected with 10 nmol/L siRNAs or scrambled control siRNA or control plasmid (pcDNA3.1 empty vector) or pcDNA3.1-ELAVL2 plasmid using Lipofectamine™ 3000 Transfection Reagent (100022052, Invitrogen) according to the manufacturer’s protocol. The efficiency of siRNAs was detected by RT-qPCR and immunoblotting.

### RNA stability assay and m6A RNA methylation quantification

To detect RNA stability, GBM cells were incubated with 10 μg/mL of Actinomycin D (A9415, Sigma-Aldrich) for 0, 4, 8, 12, 16 and 24 h. After incubation, cells were collected and RNAs were isolated. Isolated RNAs were synthesized to cDNA which was used to perform quantitative real-time PCR as manufacturer’s protocol. Quantification of m6A methylation was performed using the EpiQuik m6A RNA Methylation Quantification Kit (Colorimetric) (#P-9005, Epigentek). In this assay, total RNA was immobilized onto strip wells using a high-binding RNA solution. The presence of m6A was detected through the use of capture and detection antibodies. To enhance the detected signal, colorimetric quantification was achieved by measuring absorbance at 450 nm in a microplate spectrophotometer (Varioskan LUX Multimode Microplate Reader, Thermo). The quantity of m6A was determined proportionally based on the measured optical density (OD) intensity.

### RNA pull-down and immunoprecipitation (RIP) assay

For the RNA pull-down assay, cell lysate was mixed with biotinylated RNA, followed by the addition of streptavidin agarose beads. The binding reaction was allowed to proceed for 1 h at room temperature. The beads underwent three washes with boiled SDS buffer, and the obtained proteins were identified through western blot analysis. In the case of RIP assays, we utilized the Magna RIP™ RNA-Binding Protein Immunoprecipitation Kit (17–700, Millipore) following the manufacturer’s instructions. Briefly, cells at ~80% confluence were lysed using complete RIP lysis buffer, which included RNase and protease inhibitors. The whole cell extract was then incubated with RIP buffer containing magnetic beads conjugated to specific antibodies. The negative control involved normal mouse anti-IgG antibody (sc-3878, Santacruz), while the positive control featured the anti-SNRNP70 antibody (29–302, ProSci, Inc).

### Immunoblotting

Proteins were extracted from cells using PRO-PREP™ Protein Extraction Solution (17081, iNtRON Biotechnology). Lysates were centrifuged at 13,200 rpm for 10 min. Protein concentration was measured by Bradford assay (Bio-Rad). 20 ug of protein samples were loaded into SDS-PAGE gels (40 ug of protein samples were used for Fig. 7f to allow clearer visualization) and separated by electrophoresis, transferred onto polyvinylidene difluoride membranes (Amersham Protran), and blocked with 5% bovine serum albumin. Blots were probed with primary antibodies overnight at 4 °C. Immunoreactive bands were visualized using horseradish peroxidase-conjugated affinity purified secondary antibodies and ECL solution (1705061, Bio-rad). All antibodies were diluted to concentrations between 1:500 and 1:10,000. Immunoblotting against β-actin (sc81178, Santacruz) served as a loading control. All blots shown in each relevant panel originated from the same experiment and were processed in parallel. Uncropped scans of the immunoblots are provided in Supplementary Fig. 12. The images were captured with the ChemiDoc Touch Imaging System (Bio-Rad) and analyzed using Image Lab software. The following primary antibodies were used: anti-ELAVL2 (PA5-36157, Thermo), anti-ZEB1(ab124512, Abcam), anti-N-cadherin (ab18203, Abcam), anti-MMP3 (ab53015, Abcam), anti-CD44 (ab157107, Abcam), anti-αSMA (ab5694, Abcam), anti-IL6 (PA5-118007, Thermo), anti-SERPINE1 (MA1-40224, Thermo), anti-CCN2 (PA5-32193, Thermo), anti-SH3GL3 (HPA039381, ATLAS ANTIBODIES) and anti-DNM3(LS-C409118, LSBio). All secondary antibodies were purchased from Vector Laboratories (PI-1000 and PI-2000).

### Immunocytochemistry

Cells were seeded on coverslips 24 h prior to the experiment in 4-well plates. Cells were then rinsed twice with PBS and fixed with 0.5 ml/well of 4% paraformaldehyde/PBS (4% PFA) for 10 min at room temperature. After washing with PBS, they were permeabilized with 0.25% Triton X-100/PBS for 15 min. Cells were washed again with PBS and blocked with 0.5 ml/well of 5% normal goat serum/PBS (ab7481, Abcam) for 1 h at room temperature. Next, cells were incubated with primary antibody overnight at 4 °C, washed 3 times with PBS, and incubated with secondary antibody at room temperature. The following primary and secondary antibodies were used: anti-E-cadherin (14-3249-82, Thermo), anti-Vimentin (ab137321, Abcam), and Alexa Fluor 488- (A-11006), or 594-conjugated (A-11012) secondary antibodies (Thermo). After washing with PBS, the coverslips were mounted on a slide using VECTASHIELD Hardset antifade mounting medium with DAPI (H-1500-10, Vector Laboratories) and imaged using LSM 800 fluorescent microscope (ZEISS).

### Tissue microarray and immunohistochemistry

Glioma tissue microarrays (TMA1573, 2248, 2249, 2758) were obtained from the department of Pathology at Seoul National University Hospital (Seoul, South Korea). The TMA slides were stained using the Discovery XT automated immunohistochemistry (IHC) stainer (Ventana Medical Systems) according to the manufacturer’s instructions. Anti-ELAVL2 (PA5-36157, Thermo) was diluted 1:300. Detection was done using the Ventana Chromo Map Kit. The staining intensity of ELAVL2 was scored on a scale from 0 to 4+ based on the percentage of stained cells: score 0 for <10%, score 1+ for 10–25%, score 2+ for 25–50%, score 3+ for 50–75%, and score 4+ for >75%. Patients with scores of 0, 1 + , or 2+ were considered ELAVL2-low, while those with scores of 3+ or 4+ were considered ELAVL2-high. To compare ELAVL2 expression levels between grade II, III, and grade IV gliomas, the ELAVL2 intensity score was multiplied by 2.5 to generate expression scores on a scale of 10.

### GBM public data acquisition

Gene alteration frequency and copy number data of ELAVL1-4 in TCGA glioma patients were obtained and analyzed at various clinical features through cBioPortal online platform (https://www.cbioportal.org). Pan-cancer dataset of whole genomes (ICGC/TCGA, Nature 2020) in cBioPortal was also used to assess the ELAVL2 alteration frequency across various cancer datasets. Following gene expression datasets and clinical information of glioma patients were obtained from GlioVis (http://gliovis.bioinfo.cnio.es)^58^ to analyze ELAVL2 mRNA expression levels at various features of glioma: TCGA GBMLGG, CGGA, Ivy GBM, and TCGA U133a. Additionally, GSE53733 and GSE16011 datasets were acquired from the Gene Expression Omnibus (GEO) database to perform bioinformatics analyses.

### Bioinformatics analysis

After excluding IDH mutant samples and those with ‘NA’ for IDH status, we focused on primary IDH-wildtype GBM patients from the level 3 Affymetrix U133a microarray dataset (*N* = 357). We divided these patients into ELAVL2-high (*N* = 90) and ELAVL2-low (*N* = 90) groups, each comprising the top and bottom 25% of ELAVL2 mRNA expression levels. The same approach was used to identify ELAVL2-high and -low GBM patients in GSE16011 and GSE53733 datasets. To perform pathway analysis on ELAVL2-high and -low groups, parametric analysis of gene set enrichment (PAGE), implemented in PGSEA R package (v.1.60.0), was performed using Hallmarks (HM) and Gene Ontology (GO) gene sets obtained from the Molecular Signatures Database (MSigDB)^59^. Gene set enrichment analysis (GSEA) (www.broadinstitute.org) was performed on the desktop version of GSEA (v.4.2.0)^60^. Following gene sets were obtained from the MSigDB: Hallmark Epithelial-Mesenchymal Transition (HM_EMT), Verhaak Glioblastoma Mesenchymal (TCGA_GBM_Mesenchymal (2010)), and Verhaak Glioblastoma Proneural (TCGA_GBM_Proneural (2010)). Glioma-intrinsic gene signatures of PN (GI_Proneural (2017)) and MES (GI_Mesenchymal (2017)) subtypes were obtained from the supplementary information of Wang, Q. et al. ^4^. In GSEA, the signal-to-noise ratio was applied using 1000 permutations. The statistical significance was determined using the normalized enrichment score (NES) and *p*-value < 0.05. We also performed the weighted gene co-expression network analysis (WGCNA) using the WGCNA R package (v.1.72-1), as described in http://pklab.med.harvard.edu/scw2014/WGCNA.html^61^. The networks were computed at a soft power value of 5 and the number of top genes and minimum number of module size were set to 1000 and 20, respectively. Dynamic tree cut method was used to identify co-expression gene modules. Gene correlation analyses on GBM samples from TCGA U133a, GSE53733, and GSE16011 datasets were carried out on R2 Genomics Analysis and Visualization Platform (http://r2.amc.nl) at R correlation *p*-value ≤ 0.0001, and FDR was applied as multiple testing correction method.

### Differential expression analysis of TCGA GBM cohort

Principal component analysis (PCA) was performed on the normalized transcriptomic data of ELAVL2-high and -low GBM patients from TCGA U133a microarray dataset and was plotted using the 500 most variable genes from the whole transcriptome. Differential gene expression analysis was performed using the limma R package (v.3.52.4)^62^. A 1.5- fold change threshold with significance at *P*.adj < 0.05 was applied to define differential expression. Clusterprofiler R package (v.4.4.4) and the core analysis of Ingenuity Pathway Analysis (IPA; QIAGEN, v.23.0) were utilized to conduct functional enrichment analysis on the differentially expressed gene (DEG) profile of the ELAVL2-high GBM patients^63^. Within IPA, the graphical summary of the DEG profile was accessed using the default parameters.

### Statistical analysis

Statistical analyses were performed using GraphPad Prism (v.9.0), IBM SPSS Statistics (v.22.0), and R/Bioconductor programming language (v.4.2.3). Statistical differences between groups were assessed using Student’s *t*-test or chi-square test. Kaplan–Meier survival analysis using the log-rank statistic was conducted employing the ‘surv_cutpoint()’ function from the survminer R package (v.0.4.9), which uses maximally selected rank statistics, to determine the optimal cutoff value that best correlates with survival outcomes. All data are expressed as the mean ± standard deviation (SD). Statistical significance was defined at *P* < 0.05.

### Reporting summary

Further information on research design is available in the Nature Research Reporting Summary linked to this article.

### Supplementary information

SUPPLEMENTAL MATERIAL

REPORTING SUMMARY

## Data Availability

TCGA-GBM and -LGG datasets used in this study are publicly available in the National Cancer Institute Genomic Data Commons (GDC) Data Portal (https://portal.gdc.cancer.gov/) repository, cBioPortal (https://www.cbioportal.org) and GlioVis (http://gliovis.bioinfo.cnio.es) online platforms. Other public GBM datasets used are available through the GEO under the accession codes GSE53733 and GSE16011.

## Code availability

The R/Bioconductor programming language (v.4.2.3) and IPA (v.23.0) were employed for all bioinformatic analyses conducted in this study. Details such as packages, parameters, and versions used are described in the Methods section. The custom code used in the generation or analysis of datasets is available from the corresponding author upon request.
