# Supplementary material for: ELAVL2 loss promotes aggressive mesenchymal transition in glioblastoma
Source: NPJ Precis Oncol. 2024 Mar 28;8:79. doi: 10.1038/s41698-024-00566-1 (PMC10978835; doi:10.1038/s41698-024-00566-1)
Supplement: Supplementary file 1 — SUPPLEMENTAL MATERIAL [file 41698_2024_566_MOESM1_ESM.pdf]

Supplementary information

ELAVL2 loss promotes aggressive mesenchymal transition in glioblastoma

Yona Kim, Ji Hyeon You, Yeonjoo Ryu, Gyuri Park, Urim Lee, Hyo Eun Moon, Hye Ran Park, Chang W. Song, Ja-Lok Ku, Sung-Hye Park and Sun Ha Paek

Contents

1. Supplementary Figures -- 2

2. Supplementary Tables -- 17

Supplementary Fig. 1

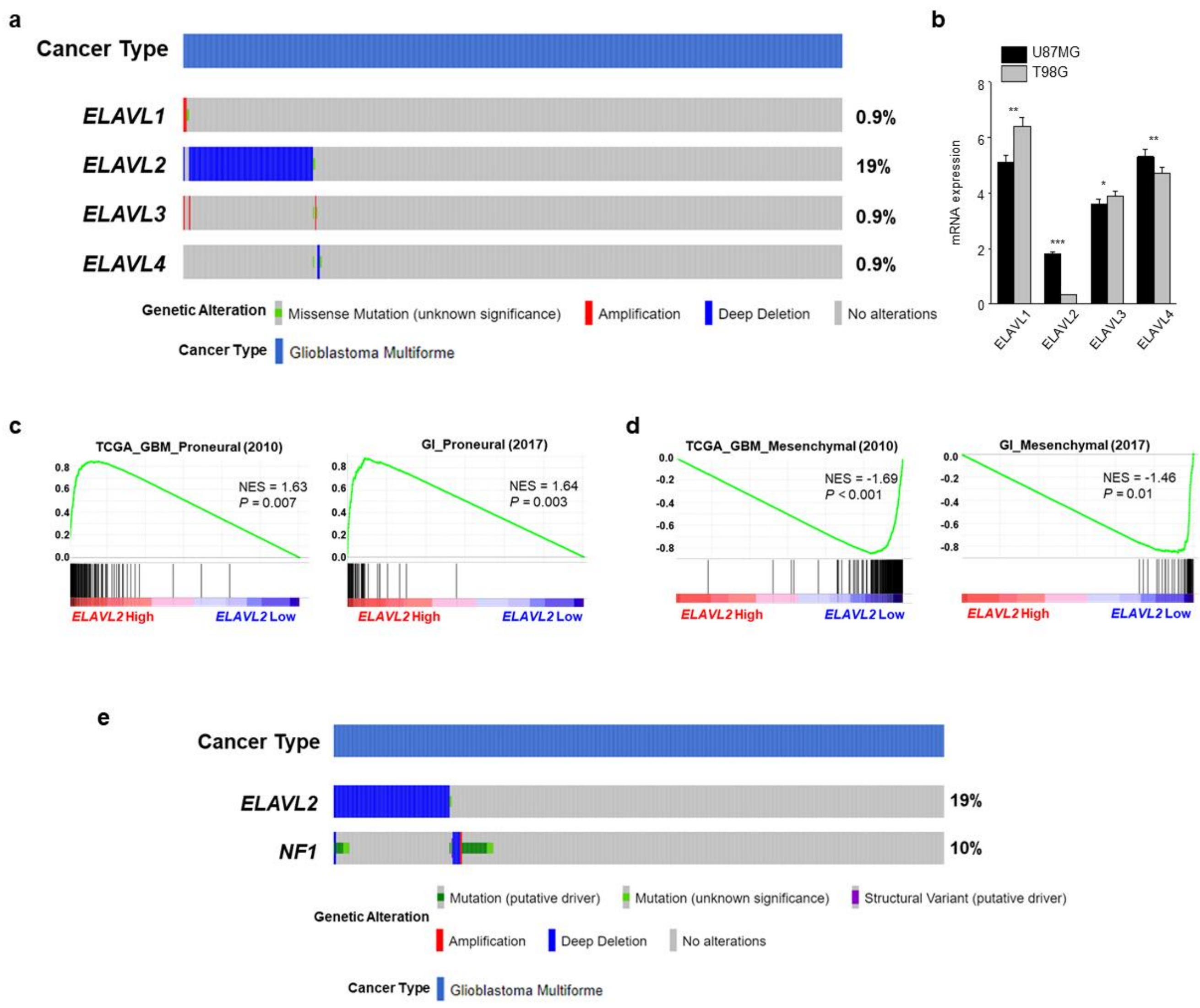

Supplementary Fig. 1 ELAVL2 is most deleted in GBM and its expression level is associated with distinct transcriptomic signature. a Copy number alteration (CNA) status of ELAVL family members (ELAVL1-4) in GBM is shown. The dataset was obtained from TCGA GBM cohort (TCGA, PanCancer Atlas) in cBioPortal platform. **b** RT-qPCR analysis of the ELAVL family members in GBM cell lines. **c-d** PN subtype was found to be significantly enriched in ELAVL2-high GBM patients **c**, whereas MES subtype was enriched in ELAVL2-low GBM patients **d**. **e** CNA status of ELAVL2 and NF1 in GBM is shown. The same dataset was used as in **a.** TCGA, The Cancer Genome Atlas; GI, Glioma-intrinsic. Years next to each gene set indicate the year in which the corresponding gene sets were established. Normalized enrichment score (NES), as well as p value, are shown. Details regarding the gene sets are provided in Materials and Methods.

Supplementary Fig. 2

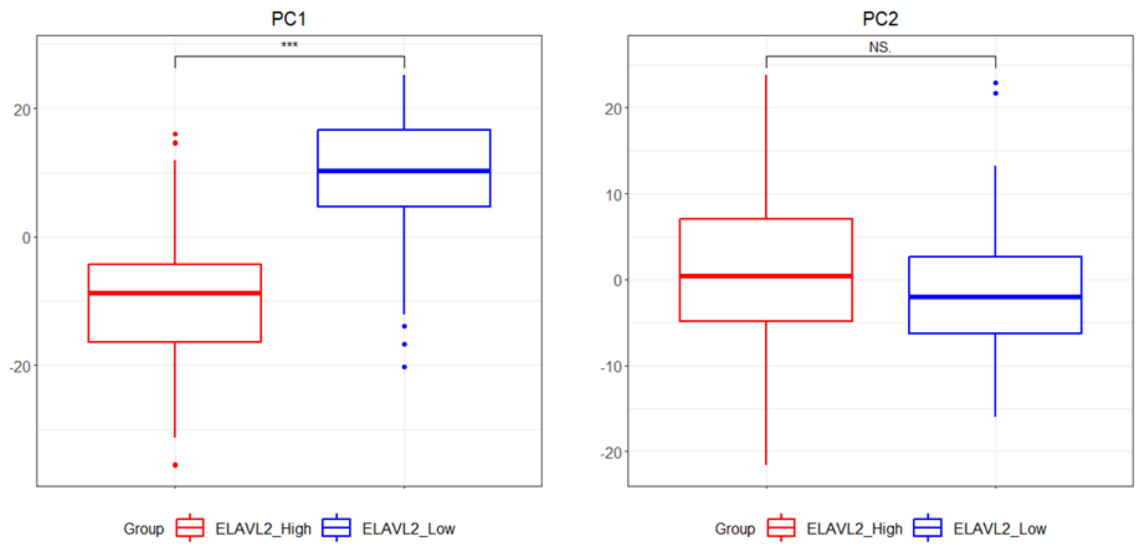

Supplementary Fig. 2 Box plots indicate that there is a significant difference in the transcriptomic profiles between ELAVL2-high and ELAVL2-low GBM patients along the PC1 dimension.

Supplementary Fig. 3

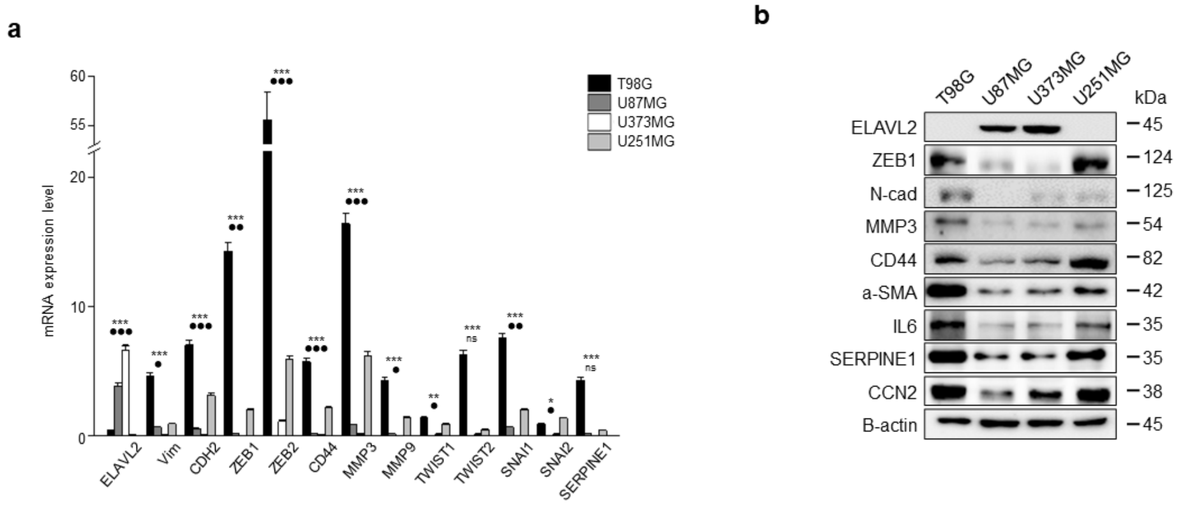

Supplementary Fig. 3 An inverse expression pattern of ELAVL2 and MES molecules in commercial GBM cell lines. a-b RT-qPCR **a** and immunoblotting **b** of ELAVL2 and MES markers were performed on GBM cell lines (T98G, U87MG, U373MG, U251MG). All data represent the mean ± SD (*n* = 3). *p < 0.05, **p < 0.01, ***p <0.001 between ELAVL2-high GBM (U87MG) and each of ELAVL2-low GBMs (T98G or U251MG). NS indicates statistically non-significant, •p < 0.05, ••p < 0.01, •••p <0.001 between ELAVL2-high GBM (U373MG) and each of ELAVL2-low GBMs (T98G or U251MG).

Supplementary Fig. 4

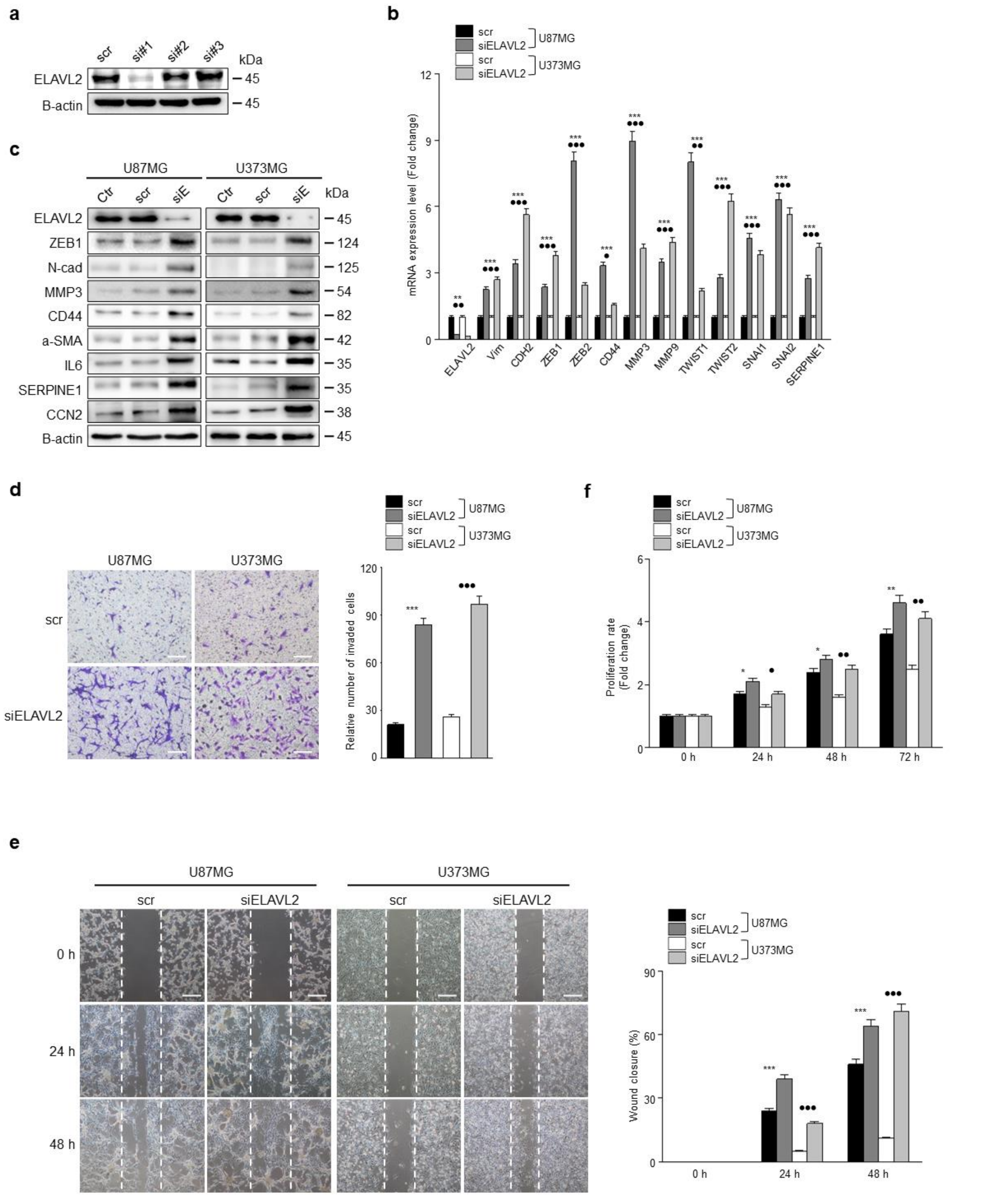

Supplementary Fig. 4 Inhibition of ELAVL2 increases MES traits in GBM cell lines. a Immunoblotting of ELAVL2 in GBM14 after ELAVL2 genetic silencing using 3 siRNA candidates. **b** mRNA levels of ELAVL2 and MES markers in U87MG and U373MG transfected with scr or siELAVL2. All data represent the mean ± SD (n = 3). **p < 0.01, ***p <0.001 between U87MG with scr transfected and U87MG with siELAVL2 transfected. •p < 0.05, ••p < 0.01, •••p <0.001

between U373MG with scr transfected and U373MG with siELAVL2 transfected. **c** Protein levels of ELAVL2 and MES-related molecules in U87MG and U373MG control (ctr) and transfected with scr or siELAVL2. **d-f** Invasion (original magnification, × 100. Scale bar, 100 μm) **d**, wound healing (original magnification, × 40. Scale bar, 250 μm) **e**, and proliferation assays **f** in U87MG and U373MG transfected with scr or siELAVL2. Representative images and bar graphs of relative wound closure **d** and invasion **e** are shown. All data represent the mean ± SD (*n* = 3). *p < 0.05, **p < 0.01, ***p <0.001 between U87MG with scr transfected and U87MG with siELAVL2 transfected. •p < 0.05, ••p < 0.01, •••p <0.001 between U373MG with scr transfected and U373MG with siELAVL2 transfected.

Supplementary Fig. 5

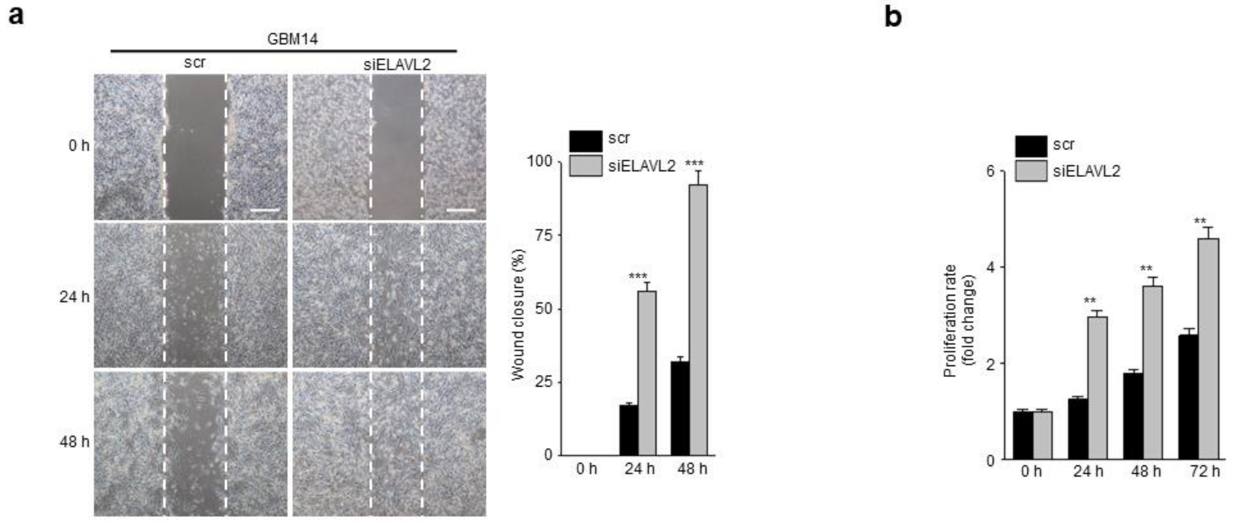

Supplementary Fig. 5 ELAVL2 inhibition increases MES characteristics in primary GBM cell lines. GBM14 was transfected with scr or siELAVL2 and subjected to wound healing **a** (original magnification, × 40. Scale bar, 250 µm) and proliferation assays **b**. Representative images and bar graphs of relative wound closure **a** are shown. All data represent the mean ± SD (*n* = 3). **p < 0.01, ***p <0.001 versus the scr group.

Supplementary Fig. 6

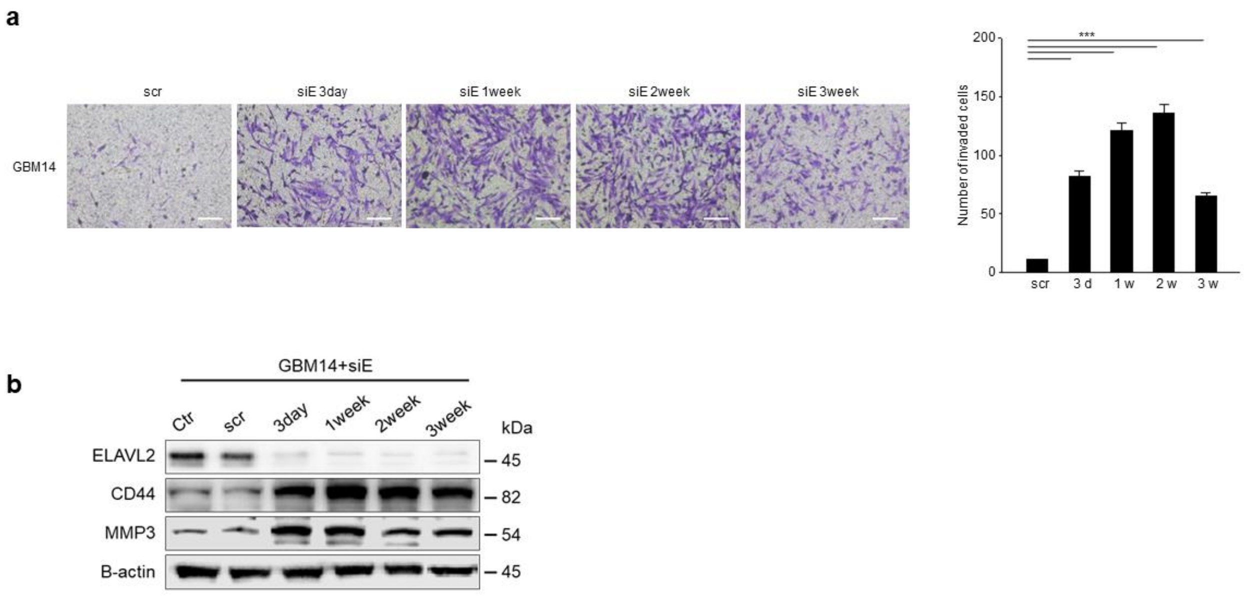

Supplementary Fig. 6 Plasticity of the MES traits after the transient silencing of ELAVL2 in GBM cell lines. a GBM14 cells were treated with siELAVL2 for different durations (3 days, 1 week, 2 weeks, and 3 weeks), and then collected for the invasion assay (original magnification, × 100. Scale bar, 100 μm). All data represent the mean ± SD (*n* = 3) ***p <0.001 versus the scramble (scr) group. Bar graph of the relative cell invasion is shown. **b** Immunoblotting of ELAVL2 and MES-related molecules in GBM14 cells transfected with either scr or siELAVL2 for the indicated durations.

Supplementary Fig. 7

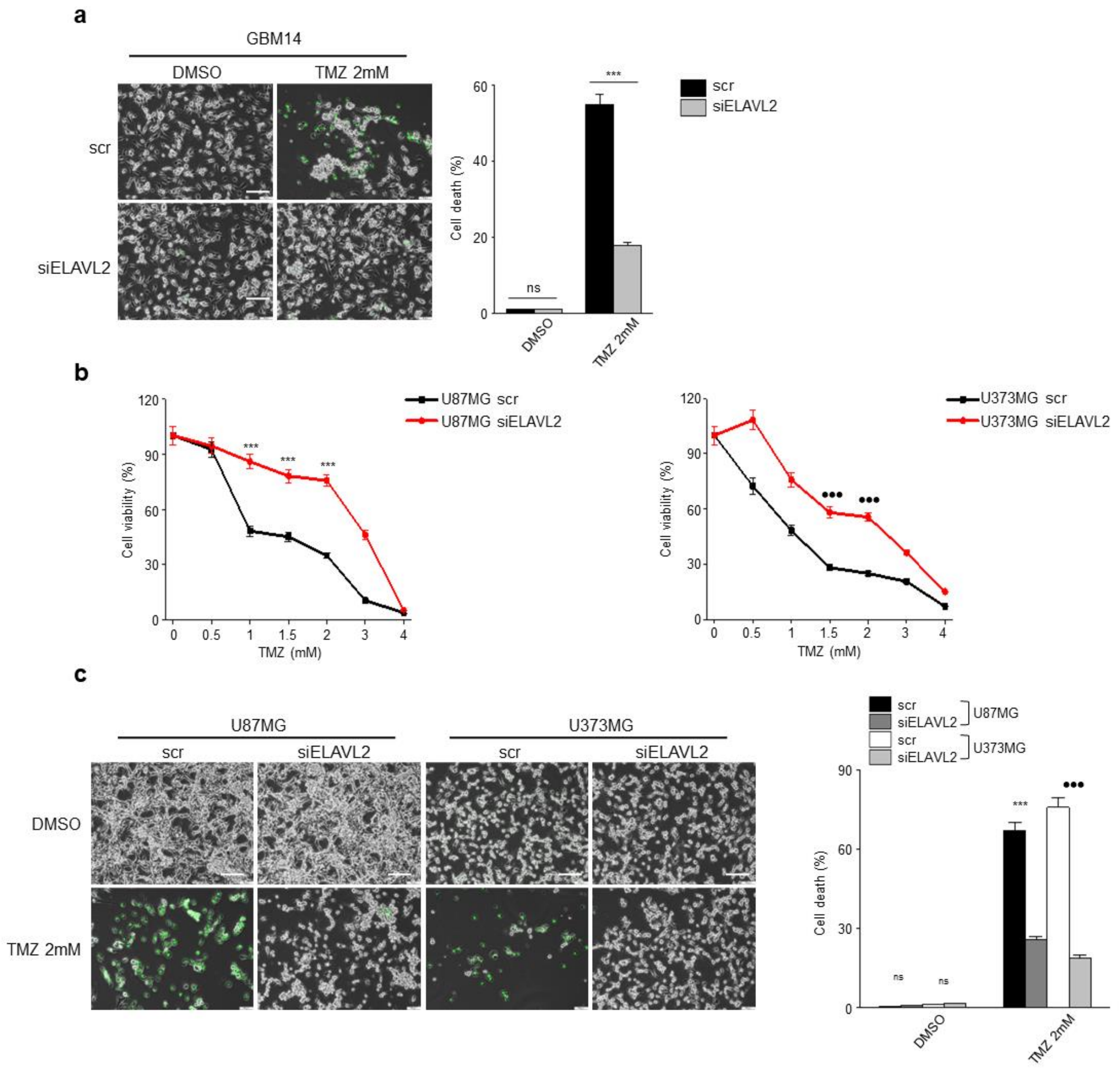

Supplementary Fig. 7 ELAVL2 down-regulation induces TMZ resistance in GBM cell lines. a Cell death assay of GBM14 transfected with scr or siELAVL2 and treated with DMSO or 2 mM TMZ for 24 h. Original magnification, × 100. Scale bar, 100 μm. NS indicates statistically non-significant, ***p <0.001 versus the scr group. **b, c** Cell viability assay **b** and cell death assay **c** of U87MG (left panel) and U373MG (right panel) transfected with scr or siELAVL2 and treated with indicated doses of TMZ for 24 h. TMZ-treated cells were normalized to DMSO control. Original magnification, × 100. Scale bar, 100 μm. All data represent the mean ± SD (*n* = 3). NS indicates statistically non-significant, ***p <0.001 between U87MG with scr transfected and U87MG with siELAVL2 transfected. •••p <0.001 between U373MG with scr transfected and U373MG with siELAVL2 transfected.

Supplementary Fig. 8

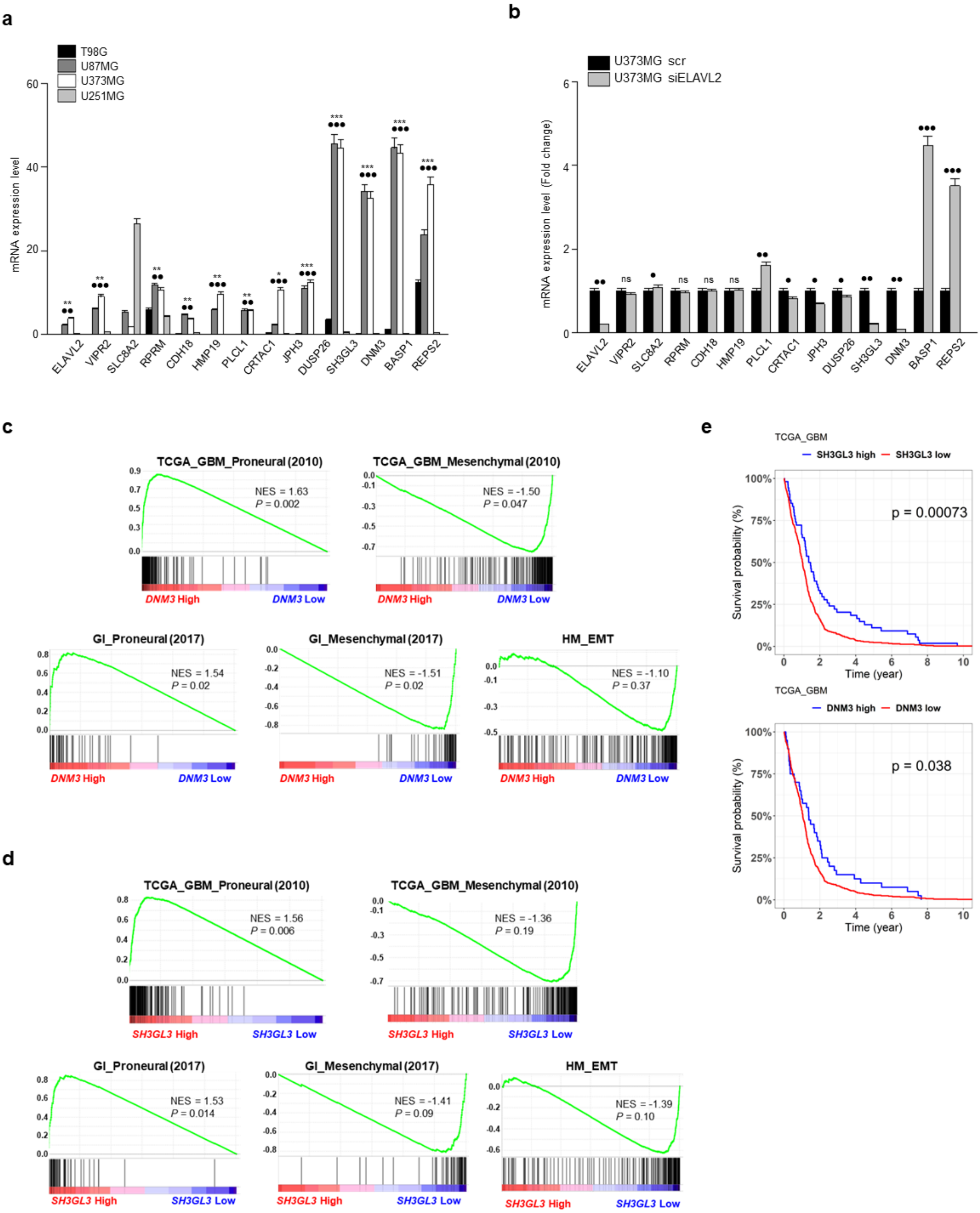

Supplementary Fig. 8 Positive correlations of ELAVL2 with SH3GL3 and DNM3, which are associated with favorable prognostic factors. a mRNA levels of ELAVL2 and 13 candidate target genes in GBM cell lines (T98G, U87MG, U373MG, and U251MG). All data represent the mean ± SD (n = 3). *p < 0.05, **p < 0.01, ***p <0.001 between ELAVL2-high GBM (U87MG) and each of ELAVL2-low GBMs (T98G or U251MG). ••p < 0.01, •••p <0.001 between ELAVL2-high GBM (U373MG) and each of ELAVL2-low GBMs (T98G or U251MG). **b** RT-qPCR of

ELAVL2 and 13 candidate target genes in U373MG treated with scr or siELAVL2. All data represent the mean ± SD (*n* = 3). NS indicates statistically non-significant, •$p < 0.05$, ••$p < 0.01$, •••$p < 0.001$ versus the scr group. **c, d** GSEA graphs of the indicated gene sets comparing DNM3-high versus DNM3-low GBM patients **c** and SH3GL3-high versus SH3GL3-low GBM patients **d** from TCGA GBM U133a microarray dataset. Normalized enrichment score (NES), as well as p value, are shown. **e** Kaplan-Meier survival curves of GBM patients from TCGA GBM U133a microarray dataset based on their SH3GL3 (upper panel) or DNM3 (lower panel) mRNA expression levels. p, log-rank test.

Supplementary Fig. 9

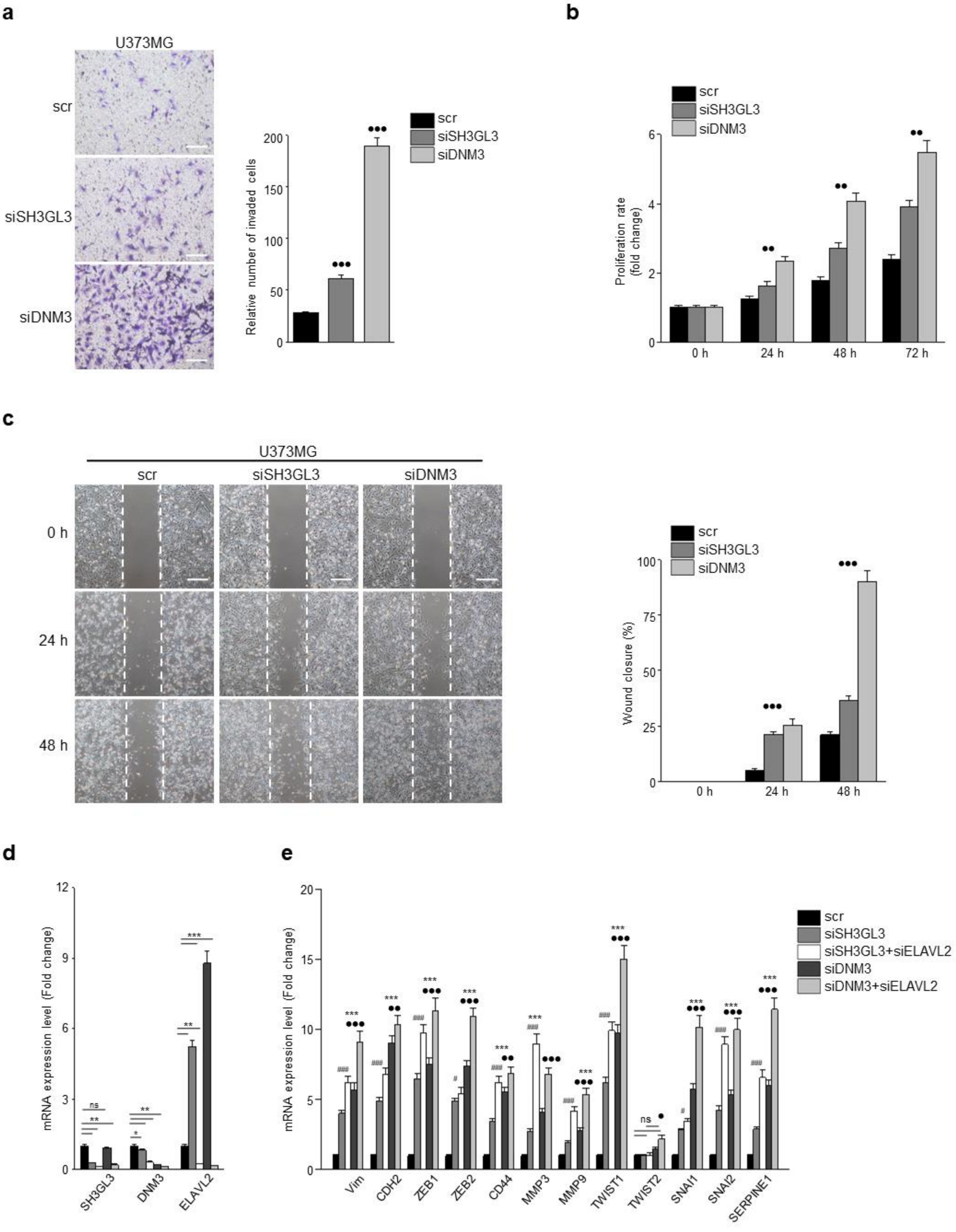

Supplementary Fig. 9 Suppression of SH3GL3 or DNM3 increases MES characteristics in GBM cells. a-c Invasion (original magnification, × 100. Scale bar, 100 μm) **a**, proliferation **b** and wound healing assays (original magnification, × 40. Scale bar, 250 μm) **c** in U373MG treated with scr or siSH3GL3 or siDNM3. Representative images and bar graphs

of relative cell invasion **a** and wound closure **c** are displayed. All data represent the mean ± SD (*n* = 3). ••p < 0.01, •••p <0.001 versus the scr group. **d, e** mRNA levels of SH3GL3, DNM3, ELAVL2 **d** and MES markers **e** in U373MG transfected with the following conditions: scr, siSH3GL3, siSH3GL3 + siELAVL2, siDNM3, and siDNM3 + siELAVL2. All cells were normalized to scr. Data are presented as the mean ± SD (*n* = 3). NS indicates statistically non-significant, *p < 0.05, **p < 0.01, ***p <0.001 versus the scr group. #p < 0.05, ##p < 0.01, ###p <0.001 between siSH3GL3 and siSH3GL3 + siELAVL2 group. •p < 0.05, ••p < 0.01, •••p <0.001 between siDNM3 and siDNM3 + siELAVL2 group.

Supplementary Fig. 10

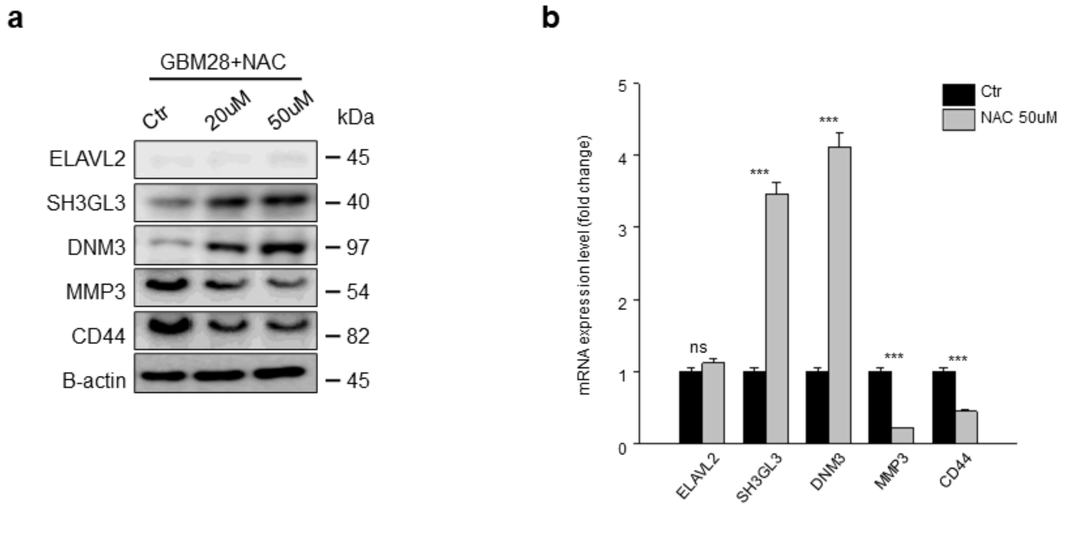

Supplementary Fig. 10 Increased expression of SH3GL3 and DNM3 leads to down-regulation of MES-related molecules in GBM cells. a-b Immunoblotting **a** and RT-qPCR **b** of ELAVL2, SH3GL3, DNM3 and the indicated MES-related molecules in GBM28 control (Ctr) and GBM28 treated with N-Acetyl cysteine (NAC) at the indicated concentrations.

Supplementary Fig. 11

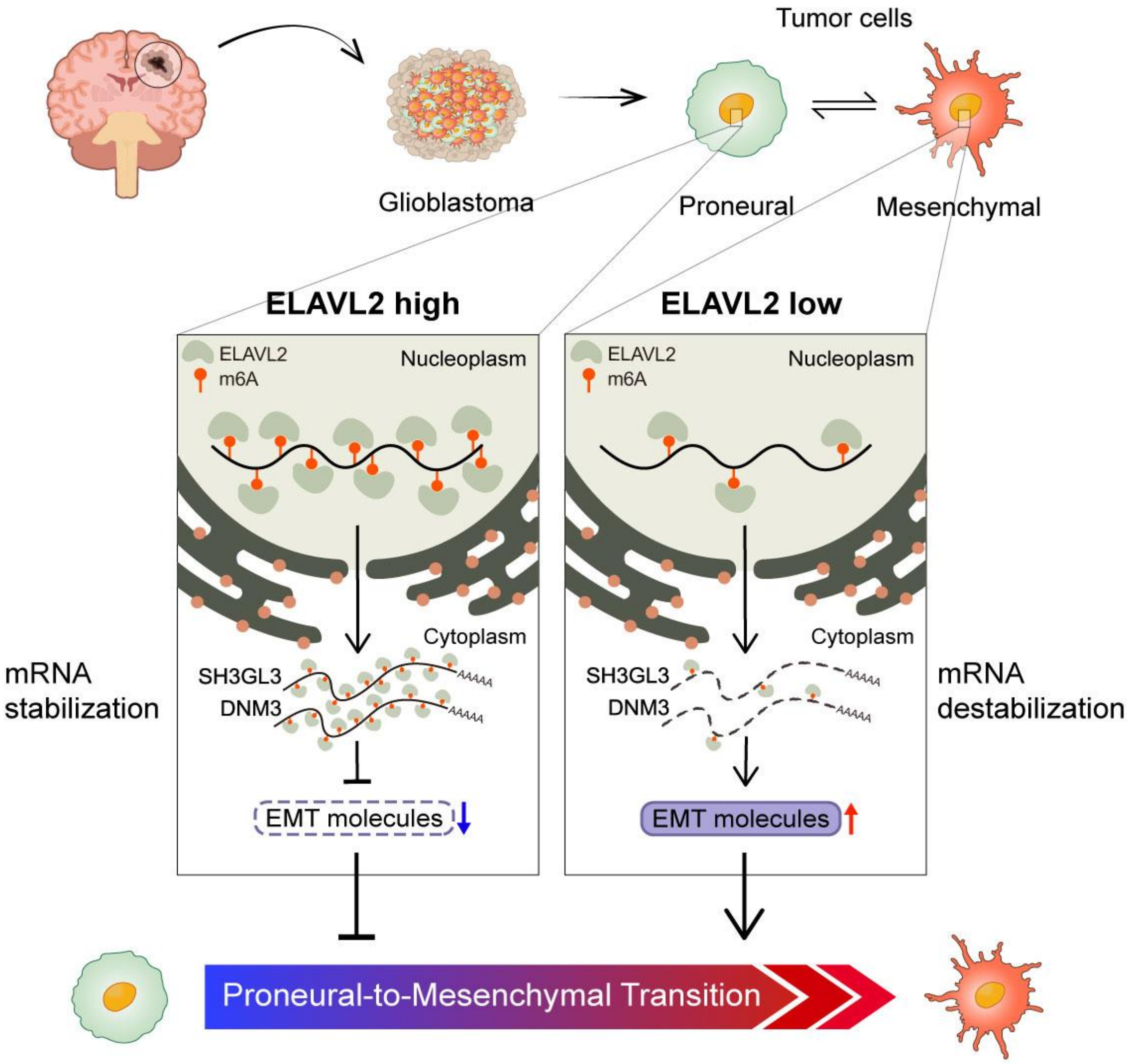

Supplementary Fig. 11 A schematic diagram illustrating the proposed mechanism of ELAVL2-mediated repression of MES-related phenotypes in GBM. ELAVL2, an RNA-binding protein, is hypothesized to directly bind to the m6A portion of target mRNA transcripts (SH3GL3 and DNM3) to regulate their mRNA stability, thereby forming a signaling axis that regulates aggressive mesenchymal transition in GBM. Within ELAVL2-high GBM cells, ELAVL2 may directly modulate downstream mRNA stability, potentially in an m6A-dependent manner. Conversely, in ELAVL2-low GBM cells, the stability of the downstream mRNA transcripts decreases, leading to increased EMT-associated phenotypes.

Supplementary Fig. 12 Uncropped images of the immunoblots

Figure 4g

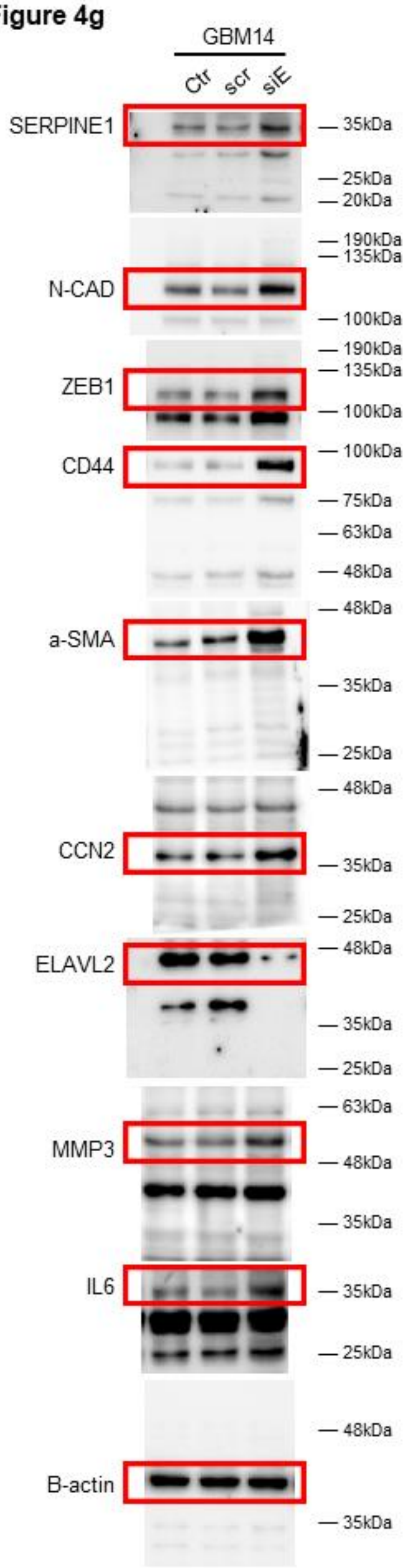

Figure 4i

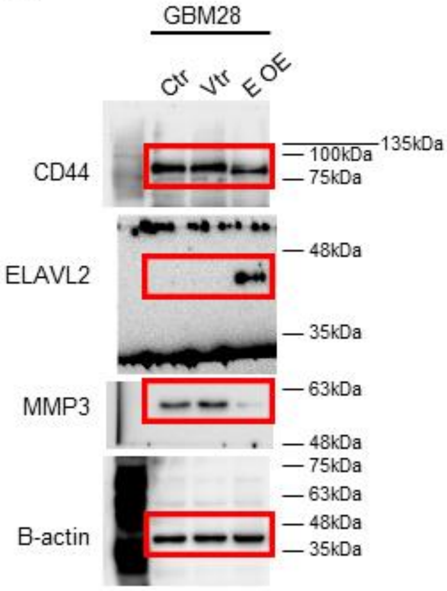

Figure 6g

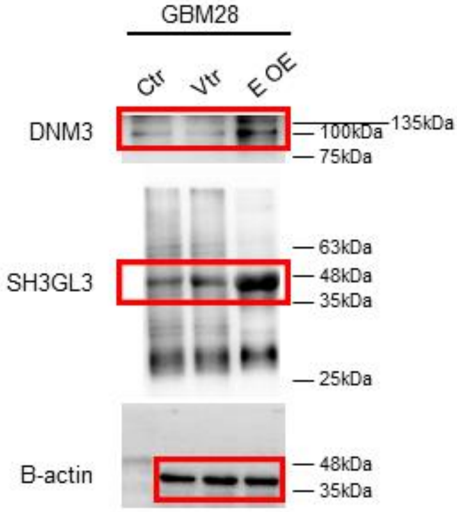

Figure 7e

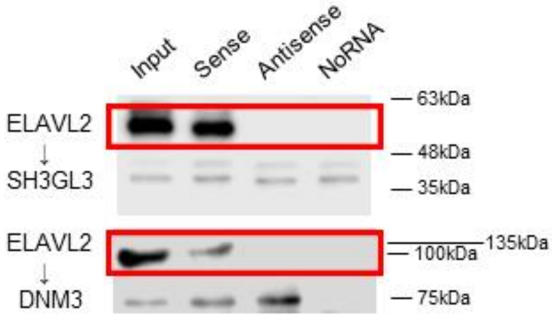

Supplementary Table 1. List of DEGs between ELAVL2-high and -low TCGA GBM patients.

Regulation	Symbol	log2 FC	Adj.Pval
Up	STMN2	3.104	2.66E-16
Up	DCX	2.762	1.36E-15
Up	PLPPR1	2.556	6.96E-12
Up	SYT1	2.134	1.55E-13
Up	MYT1L	2.094	1.20E-15
Up	ELAVL4	2.072	4.04E-14
Up	HMP19	2.062	3.42E-17
Up	SNAP91	2.018	1.17E-15
Up	INA	1.950	1.02E-15
Up	SNAP25	1.950	1.79E-12
Up	SH3GL2	1.903	3.73E-13
Up	TMSL8	1.808	1.60E-05
Up	TOX3	1.799	2.95E-10
Up	RUNDC3A	1.769	3.10E-17
Up	TUBB4	1.767	1.02E-10
Up	OPCML	1.713	8.61E-14
Up	DYNC1I1	1.705	2.82E-14
Up	KCND2	1.698	8.29E-10
Up	SOX11	1.692	5.06E-08
Up	AGXT2L1	1.688	1.89E-06
Up	PAK3	1.589	6.31E-18
Up	FGF13	1.566	1.73E-10
Up	NEFL	1.557	5.63E-07
Up	TAGLN3	1.555	9.89E-13
Up	SCN3B	1.535	5.85E-13
Up	RALYL	1.502	1.52E-12
Up	TMEM35	1.487	3.54E-15
Up	SYN1	1.474	6.64E-13
Up	CA10	1.472	1.47E-07
Up	VSNL1	1.470	1.66E-06
Up	INSM1	1.445	4.48E-06
Up	RAB33A	1.435	7.50E-10
Up	EYA1	1.425	5.13E-11
Up	ZFPM2	1.424	1.55E-07
Up	KIF21B	1.419	2.08E-13
Up	DLL3	1.400	5.33E-07
Up	NCAN	1.394	5.71E-05
Up	FGF9	1.391	1.62E-15
Up	DNM3	1.385	7.79E-12
Up	NMNAT2	1.381	5.01E-12
Up	CHGB	1.379	9.89E-13
Up	GPR17	1.376	4.56E-05
Up	CCK	1.370	9.98E-08
Up	HPCAL4	1.370	9.45E-12
Up	CHGA	1.366	2.88E-13
Up	SLC17A6	1.358	1.18E-11
Up	NEFM	1.352	1.65E-08
Up	DLX5	1.350	1.27E-06
Up	SLC17A7	1.348	1.63E-08
Up	CRYM	1.345	1.67E-08
Up	SLC12A5	1.341	2.29E-11
Up	UGT8	1.328	2.08E-06
Up	SCN3A	1.314	4.47E-06
Up	GNG3	1.305	1.48E-08
Up	STMN4	1.298	3.09E-08
Up	GNG4	1.286	9.89E-13
Up	REEP1	1.270	1.86E-09
Up	ACTL6B	1.269	3.96E-11
Up	TCEAL2	1.268	6.82E-06
Up	AMPH	1.266	1.68E-10
Up	GABRA1	1.265	5.56E-09
Up	NNAT	1.263	9.40E-05
Up	CDKN2A	1.263	6.88E-08
Up	BTBD8	1.260	1.58E-13
Up	NRGN	1.252	1.18E-07
Up	GNAO1	1.243	1.39E-16
Up	COL11A1	1.240	2.55E-04
Up	OMG	1.237	2.72E-05
Up	KIF1A	1.222	2.88E-07
Up	KCNB1	1.212	2.12E-14
Up	TMEFF1	1.212	1.72E-10
Up	DBC1	1.211	2.92E-07
Up	PPP1R1A	1.205	1.42E-10
Up	ELAVL2	1.204	1.64E-33
Up	CAMTA1	1.199	2.37E-10
Up	PLP1	1.197	1.38E-03
Up	HRASLS	1.195	1.43E-07
Up	BCL11A	1.193	7.63E-16
Up	BEX1	1.183	3.35E-05
Up	NPY	1.176	1.05E-05
Up	HPCA	1.176	5.06E-12
Up	NAP1L2	1.159	7.13E-09
Up	AK5	1.151	2.45E-08
Up	CLGN	1.147	4.68E-10
Up	NRXN1	1.147	1.06E-11
Up	GRIA2	1.146	1.52E-04
Up	ERBB3	1.145	1.84E-04
Up	TAC1	1.143	7.73E-04
Up	DGKB	1.140	4.40E-08
Up	CRABP1	1.138	4.01E-06
Up	MBP	1.132	5.77E-04
Up	SV2B	1.130	1.41E-07
Up	GABRA2	1.117	2.89E-08
Up	RPRM	1.111	1.68E-11
Up	SCG3	1.109	1.93E-04

**Up**	PKIA	1.108	2.52E-07
**Up**	GABRB1	1.103	5.95E-04
**Up**	PAK7	1.102	6.61E-12
**Up**	RASL11B	1.099	2.72E-07
**Up**	ZNF536	1.099	4.66E-10
**Up**	PCP4	1.093	4.14E-06
**Up**	NKX2-2	1.092	1.52E-04
**Up**	B4GALNT1	1.090	1.25E-06
**Up**	BASP1	1.080	1.49E-06
**Up**	CDK4	1.078	4.33E-06
**Up**	SOX10	1.075	2.08E-06
**Up**	NOL4	1.074	3.35E-08
**Up**	ATP6V1G2	1.072	7.33E-06
**Up**	FUT9	1.068	3.41E-14
**Up**	ELMO1	1.067	9.23E-08
**Up**	DDX25	1.067	8.36E-07
**Up**	PCSK2	1.062	2.82E-11
**Up**	KIF5C	1.062	8.55E-06
**Up**	TAC3	1.049	7.33E-06
**Up**	SERPINI1	1.046	2.71E-05
**Up**	BCAS1	1.040	4.68E-05
**Up**	FA2H	1.036	4.50E-06
**Up**	ZNF804A	1.035	2.60E-09
**Up**	SST	1.034	3.78E-06
**Up**	THSD7A	1.033	1.66E-06
**Up**	PEG3	1.032	2.37E-06
**Up**	EEF1A2	1.030	4.68E-05
**Up**	KLRC3	1.026	1.55E-07
**Up**	NAP1L3	1.024	5.20E-07
**Up**	CNTN1	1.021	6.87E-07
**Up**	LY6H	1.021	6.63E-06
**Up**	KLHL9	1.015	1.49E-07
**Up**	KCNE1L	1.014	3.97E-06
**Up**	PROM1	1.014	1.01E-03
**Up**	GRM3	1.012	1.72E-06
**Up**	PHACTR1	1.010	1.95E-09
**Up**	ST18	1.007	8.19E-06
**Up**	FXYD7	1.005	6.18E-06
**Up**	ATP1A3	1.002	2.70E-12
**Up**	SOX4	1.000	1.15E-07
**Up**	MAGI1	0.999	4.96E-11
**Up**	DAAM2	0.996	1.79E-05
**Up**	FGF12	0.994	3.53E-09
**Up**	WASF1	0.994	2.56E-07
**Up**	CDC7	0.992	1.79E-08
**Up**	DUSP26	0.992	1.23E-14
**Up**	ASCL1	0.985	5.13E-07
**Up**	CYP27B1	0.980	4.11E-06
**Up**	ATP10B	0.979	1.08E-05
**Up**	HOXA2	0.979	3.94E-07
**Up**	TSPAN31	0.969	1.01E-04

**Up**	MYOT	0.968	9.64E-08
**Up**	KCNK1	0.957	5.90E-06
**Up**	FAM5C	0.957	4.88E-04
**Up**	BCAN	0.955	1.39E-05
**Up**	KIT	0.948	2.75E-06
**Up**	CXORF1	0.946	7.30E-10
**Up**	CELSR3	0.942	9.39E-10
**Up**	SNCB	0.941	2.19E-08
**Up**	NMU	0.938	5.30E-05
**Up**	MOG	0.936	2.20E-03
**Up**	NELL2	0.928	1.41E-04
**Up**	TOP2A	0.927	2.47E-05
**Up**	GABBR1	0.923	1.35E-05
**Up**	LRRTM4	0.919	2.85E-09
**Up**	TSFM	0.918	3.07E-05
**Up**	PTBP2	0.915	2.58E-09
**Up**	METTL1	0.915	8.09E-05
**Up**	MSTN	0.914	9.17E-05
**Up**	NAV3	0.912	3.17E-06
**Up**	RNF144A	0.911	1.52E-07
**Up**	SATB1	0.909	9.23E-08
**Up**	OLFM1	0.908	5.03E-07
**Up**	NTSR2	0.907	1.64E-04
**Up**	SCN2A	0.904	3.47E-12
**Up**	NUDT11	0.902	2.65E-06
**Up**	CDK5R1	0.901	6.58E-14
**Up**	CRMP1	0.899	5.99E-05
**Up**	PDZRN4	0.899	6.60E-09
**Up**	CXORF57	0.895	2.72E-04
**Up**	KLHL23	0.895	7.67E-11
**Up**	SYNGR3	0.895	2.37E-06
**Up**	GDAP1L1	0.890	1.16E-09
**Up**	PPP1R16B	0.889	1.31E-07
**Up**	NRXN2	0.886	1.19E-07
**Up**	RAPGEF4	0.885	3.55E-06
**Up**	ZNF423	0.884	1.46E-05
**Up**	KCNJ16	0.883	1.74E-02
**Up**	FAM130A2	0.882	4.96E-11
**Up**	CDH18	0.882	3.39E-13
**Up**	CSPG5	0.881	2.11E-03
**Up**	RIT2	0.879	4.69E-03
**Up**	A2BP1	0.878	1.98E-10
**Up**	EPHB1	0.876	2.08E-06
**Up**	BSN	0.872	2.48E-13
**Up**	CRB1	0.871	1.41E-06
**Up**	CXXC4	0.870	2.06E-08
**Up**	FAM119B	0.869	1.53E-03
**Up**	MOBP	0.868	1.09E-03
**Up**	SUSD4	0.867	6.44E-07
**Up**	RIMBP2	0.852	1.36E-07
**Up**	PCSK1N	0.852	6.80E-04

**Up**	C6ORF134	0.851	1.27E-05
**Up**	SLC38A1	0.850	1.92E-04
**Up**	DTL	0.844	7.40E-06
**Up**	PRKAR2B	0.841	1.47E-06
**Up**	STMN1	0.838	1.87E-11
**Up**	MPPED2	0.837	1.69E-07
**Up**	FHOD3	0.834	1.10E-08
**Up**	RTN1	0.830	6.31E-03
**Up**	TTK	0.830	1.54E-05
**Up**	FAM77C	0.828	1.17E-12
**Up**	PDYN	0.825	5.78E-06
**Up**	PCLO	0.821	5.35E-12
**Up**	MAD2L1	0.818	1.72E-05
**Up**	CAMKV	0.816	1.46E-11
**Up**	MAPT	0.816	1.58E-05
**Up**	DIRAS2	0.813	4.11E-03
**Up**	ARPP21	0.813	6.55E-06
**Up**	WIF1	0.810	5.55E-03
**Up**	SCN1A	0.809	1.10E-05
**Up**	C20ORF42	0.809	1.39E-03
**Up**	STXBP1	0.808	1.39E-05
**Up**	NBEA	0.808	4.91E-05
**Up**	NEFH	0.808	8.96E-07
**Up**	BCL7A	0.807	3.65E-10
**Up**	DGKI	0.807	6.98E-08
**Up**	PTPRD	0.806	1.37E-06
**Up**	PHYHIP	0.805	5.65E-09
**Up**	HNT	0.804	1.24E-05
**Up**	RAI2	0.804	7.18E-08
**Up**	C20ORF103	0.804	2.46E-05
**Up**	POU4F1	0.803	1.92E-06
**Up**	FLRT1	0.799	7.56E-09
**Up**	TMEM16C	0.799	5.39E-10
**Up**	CLSTN2	0.797	4.63E-08
**Up**	GNAI1	0.795	2.99E-04
**Up**	SSBP3	0.792	1.54E-07
**Up**	ALDOC	0.788	1.84E-03
**Up**	TSPAN12	0.785	1.19E-03
**Up**	RAB3A	0.784	3.98E-11
**Up**	ATP1A2	0.783	4.03E-02
**Up**	PLEKHA5	0.782	2.42E-04
**Up**	SULT4A1	0.781	7.30E-10
**Up**	ENC1	0.778	1.04E-05
**Up**	NCALD	0.774	6.51E-05
**Up**	MLF1IP	0.774	1.04E-04
**Up**	MLLT11	0.773	4.06E-04
**Up**	CUTL2	0.768	3.48E-08
**Up**	CD200	0.768	5.14E-08
**Up**	B3GALT2	0.767	2.12E-07
**Up**	GABBR2	0.764	6.14E-05
**Up**	MYRIP	0.764	3.21E-05

**Up**	CIT	0.760	1.18E-07
**Up**	RIMS2	0.758	9.89E-13
**Up**	PDGFRA	0.756	2.56E-03
**Up**	MAP2	0.754	1.67E-04
**Up**	NPPA	0.751	2.54E-05
**Up**	KIF5A	0.750	7.41E-09
**Up**	CHD7	0.747	2.88E-05
**Up**	RASL10A	0.746	4.28E-06
**Up**	MAGEH1	0.746	9.86E-07
**Up**	HS3ST2	0.745	2.69E-05
**Up**	AP3B2	0.745	8.24E-08
**Up**	HSPA12A	0.744	7.42E-05
**Up**	C14orf132	0.743	2.12E-04
**Up**	TF	0.742	7.30E-02
**Up**	PIP3-E	0.741	1.56E-05
**Up**	NRIP3	0.740	1.10E-05
**Up**	CENPF	0.740	7.89E-05
**Up**	ATP1B1	0.739	1.04E-05
**Up**	SUSD5	0.738	7.76E-06
**Up**	ZNF238	0.738	3.18E-07
**Up**	PDE2A	0.737	3.10E-08
**Up**	LOC728215	0.737	1.63E-07
**Up**	MAGEL2	0.736	1.21E-11
**Up**	SH3GL3	0.736	2.76E-11
**Up**	MOBKL2B	0.735	4.46E-09
**Up**	C1ORF106	0.733	1.59E-06
**Up**	CYFIP2	0.730	2.84E-06
**Up**	ASRGL1	0.727	9.89E-07
**Up**	NTRK2	0.726	2.50E-03
**Up**	PGBD5	0.725	1.33E-05
**Up**	NHLH1	0.725	8.91E-06
**Up**	HTR2A	0.724	4.04E-08
**Up**	GADD45G	0.724	1.04E-06
**Up**	MARCKSL1	0.723	3.87E-05
**Up**	ELOVL4	0.723	3.80E-08
**Up**	ASPM	0.722	2.26E-04
**Up**	EFCBP2	0.721	3.13E-10
**Up**	SLC1A1	0.721	5.03E-06
**Up**	PPP1R9A	0.720	2.70E-05
**Up**	RGS7	0.719	1.10E-14
**Up**	SEZ6L	0.718	8.73E-05
**Up**	MN1	0.717	6.11E-05
**Up**	ANKRD46	0.716	1.52E-05
**Up**	PAIP2B	0.713	2.84E-07
**Up**	TRIM36	0.712	1.64E-04
**Up**	CLASP2	0.711	1.71E-05
**Up**	RUNDC3B	0.708	2.25E-06
**Up**	NDN	0.707	9.70E-03
**Up**	MYT1	0.707	7.22E-10
**Up**	GAD1	0.705	1.44E-05
**Up**	ITM2A	0.705	6.81E-04

**Up**	ATRNL1	0.700	3.35E-07
**Up**	MAL	0.698	1.01E-02
**Up**	RELN	0.697	4.33E-07
**Up**	GPRASP1	0.697	1.39E-04
**Up**	PID1	0.696	3.33E-04
**Up**	GPR23	0.695	5.97E-08
**Up**	DLK1	0.693	3.74E-02
**Up**	FSD1	0.692	8.63E-07
**Up**	GUCY1B3	0.691	1.40E-05
**Up**	C1ORF61	0.690	8.76E-02
**Up**	OS9	0.687	7.94E-04
**Up**	PIMREG	0.687	2.96E-04
**Up**	RAP1GAP	0.686	7.85E-05
**Up**	AMOTL2	0.685	1.05E-04
**Up**	S100A1	0.684	2.57E-04
**Up**	NELL1	0.682	2.19E-04
**Up**	DPF1	0.682	4.85E-10
**Up**	SCAMP5	0.682	2.17E-08
**Up**	MNX1	0.677	1.63E-08
**Up**	HMGB3	0.676	1.36E-07
**Up**	GABRG2	0.676	5.13E-07
**Up**	PLLP	0.676	2.80E-03
**Up**	ZEB2	0.675	2.57E-04
**Up**	APOD	0.673	5.81E-02
**Up**	LSAMP	0.673	1.20E-03
**Up**	PNOC	0.672	3.41E-04
**Up**	HOXC10	0.672	5.23E-03
**Up**	KIF15	0.670	2.85E-04
**Up**	HOXA9	0.670	1.35E-03
**Up**	CP110	0.669	1.16E-05
**Up**	SLC6A15	0.669	2.48E-07
**Up**	TSPYL4	0.667	1.39E-05
**Up**	HOXC6	0.666	4.87E-03
**Up**	CCNE2	0.666	1.44E-04
**Up**	ALCAM	0.665	3.42E-05
**Up**	SLCO1C1	0.664	1.37E-02
**Up**	GRB14	0.662	3.15E-04
**Up**	SYP	0.662	3.82E-09
**Up**	NEUROD1	0.660	1.94E-03
**Up**	SOX5	0.660	1.68E-05
**Up**	MYBL1	0.660	1.44E-04
**Up**	PFN2	0.659	7.15E-06
**Up**	PBK	0.658	3.84E-03
**Up**	TSPAN7	0.658	3.82E-03
**Up**	NCAM1	0.658	2.72E-06
**Up**	TMEM100	0.656	2.30E-02
**Up**	ZNF365	0.654	7.73E-04
**Up**	MEIS2	0.653	8.48E-03
**Up**	PTPRT	0.653	4.21E-06
**Up**	EZH2	0.652	5.80E-04
**Up**	MLLT3	0.651	5.52E-08

**Up**	PHGDH	0.650	4.86E-05
**Up**	MYO16	0.650	1.19E-05
**Up**	ENSG00000283228	0.646	5.62E-08
**Up**	SV2C	0.646	1.55E-07
**Up**	REXO5	0.646	5.01E-07
**Up**	YPEL1	0.645	3.16E-08
**Up**	FXYD6	0.645	8.19E-03
**Up**	KIAA1166	0.645	3.74E-07
**Up**	LRRTM2	0.642	1.23E-02
**Up**	CDCA8	0.641	6.87E-07
**Up**	PEG10	0.640	8.16E-03
**Up**	PPFIA2	0.635	1.49E-07
**Up**	BIRC5	0.635	1.48E-04
**Up**	EN2	0.633	1.47E-04
**Up**	IGSF1	0.632	1.47E-03
**Up**	ZNF711	0.632	3.87E-05
**Up**	FAM107A	0.630	3.99E-02
**Up**	XYLT1	0.629	2.02E-03
**Up**	ZNF167	0.628	5.89E-05
**Up**	DOCK3	0.627	3.50E-06
**Up**	RPS6KA5	0.626	6.48E-07
**Up**	ACSL6	0.625	9.34E-06
**Up**	PLCL1	0.625	1.66E-06
**Up**	ABAT	0.624	6.59E-03
**Up**	BAI3	0.620	1.28E-02
**Up**	CAPN3	0.619	8.80E-03
**Up**	IGF2	0.617	4.77E-02
**Up**	SYN2	0.615	1.32E-07
**Up**	RIMS3	0.615	5.14E-14
**Up**	ATP2B2	0.614	9.88E-10
**Up**	SLC30A10	0.613	1.22E-08
**Up**	GULP1	0.612	7.82E-03
**Up**	PTPRO	0.610	3.21E-04
**Up**	ERC2	0.610	2.92E-03
**Up**	XK	0.609	3.77E-05
**Up**	TSPAN13	0.609	3.14E-05
**Up**	EN1	0.609	7.03E-03
**Up**	BCOR	0.602	3.38E-06
**Up**	ERBB4	0.601	6.45E-05
**Up**	EHD3	0.601	6.75E-04
**Up**	DLX2	0.600	3.93E-08
**Up**	VIPR2	0.599	5.74E-07
**Up**	NR0B1	0.599	8.80E-03
**Up**	CDC20	0.598	9.33E-04
**Up**	PAFAH1B3	0.598	6.14E-05
**Up**	RACGAP1	0.597	4.39E-05
**Up**	MCM3APAS	0.597	2.33E-05
**Up**	CNTNAP2	0.597	1.09E-06
**Up**	SOX3	0.597	3.57E-04
**Up**	VASH2	0.596	5.79E-06
**Up**	STXBP6	0.593	3.20E-04

**Up**	PKP4	0.593	2.02E-04
**Up**	KIF11	0.593	7.89E-04
**Up**	ECT2	0.593	6.83E-04
**Up**	B3GAT1	0.591	4.15E-03
**Up**	ZNF124	0.590	7.88E-06
**Up**	DEPDC1	0.590	7.42E-04
**Up**	FBXO2	0.589	6.81E-05
**Up**	IL11RA	0.589	9.81E-04
**Up**	KIF4A	0.588	1.66E-04
**Up**	CACNG3	0.588	1.00E-05
**Up**	PPM1E	0.586	5.97E-08
**Up**	TMEM28	0.586	2.14E-11
**Down**	POSTN	-1.957	4.70E-05
**Down**	LTF	-1.590	1.28E-03
**Down**	CXCL14	-1.542	3.06E-05
**Down**	PLA2G2A	-1.524	3.47E-05
**Down**	PLA2G5	-1.377	5.63E-07
**Down**	ABCC3	-1.375	1.06E-10
**Down**	CHI3L1	-1.330	1.39E-04
**Down**	PTX3	-1.284	6.20E-05
**Down**	C1S	-1.250	3.48E-09
**Down**	SRPX2	-1.236	3.94E-09
**Down**	F13A1	-1.200	4.21E-04
**Down**	NNMT	-1.187	2.78E-04
**Down**	CA12	-1.160	1.61E-06
**Down**	IGFBP3	-1.121	4.89E-05
**Down**	CCL2	-1.086	2.84E-04
**Down**	SERPINE1	-1.069	7.58E-06
**Down**	EFEMP1	-1.029	4.18E-05
**Down**	PLAU	-1.017	1.33E-07
**Down**	AHNAK2	-1.013	3.30E-06
**Down**	AIM1	-1.009	8.28E-07
**Down**	LGALS3	-1.000	5.56E-08
**Down**	NAMPT	-0.997	4.16E-07
**Down**	C1RL	-0.992	6.38E-08
**Down**	PI3	-0.980	4.43E-03
**Down**	DPYD	-0.979	1.16E-06
**Down**	SLPI	-0.966	7.73E-03
**Down**	S100A10	-0.964	3.75E-07
**Down**	ADM	-0.956	1.94E-04
**Down**	EMP3	-0.953	4.78E-07
**Down**	CLEC5A	-0.943	4.03E-06
**Down**	FLJ21963	-0.942	4.93E-06
**Down**	SDC4	-0.940	2.93E-06
**Down**	CD44	-0.932	1.62E-07
**Down**	PDPN	-0.925	7.36E-05
**Down**	FER1L3	-0.921	9.08E-09
**Down**	LIF	-0.919	3.59E-07
**Down**	GBP2	-0.915	9.23E-08
**Down**	MAN1C1	-0.912	7.73E-05
**Down**	CP	-0.912	2.00E-04

**Down**	SLC22A18	-0.912	9.18E-11
**Down**	SRPX	-0.907	4.33E-04
**Down**	ANXA1	-0.890	1.06E-05
**Down**	PTRF	-0.888	2.00E-09
**Down**	CH25H	-0.880	2.08E-04
**Down**	MREG	-0.879	3.78E-06
**Down**	COL6A3	-0.878	2.80E-02
**Down**	CXCL8	-0.871	6.49E-03
**Down**	COL3A1	-0.868	8.54E-03
**Down**	PLTP	-0.859	1.30E-05
**Down**	TNC	-0.858	1.03E-04
**Down**	CHRNA9	-0.851	2.96E-04
**Down**	ARSJ	-0.844	1.25E-05
**Down**	COPZ2	-0.840	2.54E-06
**Down**	PROS1	-0.839	1.61E-06
**Down**	DKK1	-0.838	3.00E-02
**Down**	FZD7	-0.838	1.11E-05
**Down**	ALOX5AP	-0.835	6.33E-04
**Down**	COL5A1	-0.833	7.93E-04
**Down**	ANXA2	-0.833	2.13E-08
**Down**	SERPING1	-0.827	2.22E-05
**Down**	IQGAP1	-0.826	6.55E-11
**Down**	CD163	-0.811	3.50E-03
**Down**	KIAA1199	-0.810	1.10E-04
**Down**	CNIH3	-0.810	7.98E-05
**Down**	LUM	-0.806	1.95E-02
**Down**	PYGL	-0.804	1.48E-07
**Down**	S100A4	-0.803	4.70E-04
**Down**	GBP1	-0.803	1.15E-04
**Down**	TREM1	-0.799	2.29E-03
**Down**	FCGBP	-0.795	1.40E-02
**Down**	FCGR2B	-0.794	1.11E-03
**Down**	CAV1	-0.793	1.41E-03
**Down**	CHST2	-0.786	9.69E-06
**Down**	PRSS23	-0.785	3.67E-05
**Down**	SOD2	-0.782	1.18E-04
**Down**	EMP1	-0.777	4.26E-06
**Down**	STEAP3	-0.774	1.70E-05
**Down**	CCL20	-0.773	3.25E-03
**Down**	SLC39A14	-0.773	2.94E-06
**Down**	CFI	-0.772	3.99E-04
**Down**	RCAN1	-0.771	4.08E-04
**Down**	RNASE4	-0.768	1.92E-06
**Down**	MXRA5	-0.768	1.12E-02
**Down**	SERPINH1	-0.759	1.72E-06
**Down**	ACOX2	-0.758	1.16E-06
**Down**	TIMP1	-0.756	4.54E-06
**Down**	TCAG7.1314	-0.755	9.59E-07
**Down**	BHLHE40	-0.749	6.78E-06
**Down**	DRAM	-0.746	4.78E-06
**Down**	CPVL	-0.743	4.23E-05

**Down**	CASP1	-0.739	1.92E-06
**Down**	HRH1	-0.738	1.40E-05
**Down**	COL15A1	-0.738	1.40E-03
**Down**	BST2	-0.736	3.88E-04
**Down**	MOXD1	-0.733	2.63E-02
**Down**	HLA-DQA1	-0.732	8.55E-02
**Down**	TAGLN	-0.730	5.51E-03
**Down**	LAMA2	-0.726	1.41E-05
**Down**	ASPN	-0.725	8.28E-03
**Down**	MVP	-0.725	4.07E-07
**Down**	NDRG1	-0.722	1.94E-04
**Down**	MICALL2	-0.720	1.38E-09
**Down**	FLJ20273	-0.720	6.34E-05
**Down**	C8ORF4	-0.718	2.44E-04
**Down**	CXCL2	-0.718	1.65E-02
**Down**	S100A8	-0.717	1.32E-02
**Down**	PLP2	-0.715	8.09E-05
**Down**	FAM129A	-0.715	6.67E-05
**Down**	LOXL1	-0.709	9.14E-04
**Down**	SERPINA1	-0.708	3.06E-03
**Down**	ANGPTL4	-0.703	1.25E-03
**Down**	KLF6	-0.702	7.84E-07
**Down**	ANG	-0.702	5.97E-08
**Down**	TRIP6	-0.702	1.71E-06
**Down**	TUBB6	-0.701	3.39E-04
**Down**	AEBP1	-0.700	1.91E-03
**Down**	SLC2A10	-0.699	9.06E-05
**Down**	CHI3L2	-0.698	6.94E-02
**Down**	CSTA	-0.695	3.22E-03
**Down**	GADD45A	-0.693	7.07E-06
**Down**	CYBRD1	-0.690	6.54E-05
**Down**	WIPI1	-0.688	3.92E-06
**Down**	RNASE2	-0.684	2.45E-03
**Down**	ACTN1	-0.684	6.39E-05
**Down**	CD151	-0.684	8.87E-09
**Down**	MAFF	-0.683	4.36E-05
**Down**	TRIM22	-0.683	8.76E-05
**Down**	TNFRSF1A	-0.680	2.10E-08
**Down**	SERPINF1	-0.680	2.01E-03
**Down**	SYNPO	-0.676	3.54E-04
**Down**	C21orf62	-0.675	1.47E-02
**Down**	PCOLCE	-0.674	4.91E-03
**Down**	GLIPR1	-0.670	4.31E-04
**Down**	OSMR	-0.669	3.56E-05
**Down**	CD97	-0.669	2.43E-06
**Down**	IL6	-0.665	1.28E-02
**Down**	TDO2	-0.665	3.44E-02
**Down**	PLOD2	-0.662	8.76E-06
**Down**	HOPX	-0.659	1.09E-02
**Down**	S100A11	-0.658	4.59E-05
**Down**	APOBEC3G	-0.655	6.23E-05
**Down**	PCSK5	-0.655	7.89E-05
**Down**	FAM38A	-0.655	3.44E-07
**Down**	LY96	-0.654	3.09E-03
**Down**	ITGA7	-0.654	1.39E-03
**Down**	RAB20	-0.654	1.94E-06
**Down**	CASP4	-0.654	1.62E-05
**Down**	ISG20	-0.652	3.10E-06
**Down**	PSMB9	-0.651	5.14E-05
**Down**	CYP1B1	-0.651	5.43E-03
**Down**	COL5A2	-0.647	4.62E-03
**Down**	STAB1	-0.644	4.40E-04
**Down**	COL6A2	-0.644	1.64E-03
**Down**	S100A9	-0.641	1.98E-02
**Down**	CCDC109B	-0.639	5.11E-06
**Down**	MEOX2	-0.637	8.22E-02
**Down**	UPP1	-0.636	1.38E-04
**Down**	ANKRD25	-0.636	6.15E-08
**Down**	PLAUR	-0.636	1.81E-05
**Down**	GADD45B	-0.635	1.06E-05
**Down**	LAMB1	-0.635	4.74E-03
**Down**	VSIG4	-0.634	9.43E-03
**Down**	EFEMP2	-0.634	4.07E-06
**Down**	TMEM140	-0.633	1.58E-04
**Down**	MRC2	-0.632	1.14E-05
**Down**	MTMR11	-0.630	4.30E-06
**Down**	LRP10	-0.630	3.93E-08
**Down**	RRAS	-0.629	3.72E-06
**Down**	LOX	-0.626	8.85E-04
**Down**	VEGFA	-0.626	8.17E-03
**Down**	CSRP2	-0.624	1.67E-03
**Down**	TCIRG1	-0.623	8.28E-07
**Down**	ATF3	-0.622	1.16E-03
**Down**	FAS	-0.621	1.16E-04
**Down**	PTGS1	-0.618	3.58E-05
**Down**	ANXA4	-0.618	6.57E-05
**Down**	RBMS1	-0.618	4.94E-07
**Down**	TGFBI	-0.617	3.84E-03
**Down**	COL4A1	-0.617	3.22E-03
**Down**	GPX3	-0.616	2.52E-02
**Down**	PTGS2	-0.615	1.55E-02
**Down**	CCL18	-0.614	9.38E-02
**Down**	ZFP36	-0.613	6.03E-04
**Down**	TGFB1I1	-0.612	5.53E-05
**Down**	PIPOX	-0.611	3.38E-02
**Down**	LAMB2	-0.611	1.92E-05
**Down**	IER3	-0.611	1.21E-02
**Down**	YAP1	-0.610	7.34E-04
**Down**	TNFRSF12A	-0.610	2.35E-05
**Down**	SHC1	-0.610	5.28E-06
**Down**	HP	-0.610	3.47E-02
**Down**	HS3ST3A1	-0.609	5.87E-03

**Down**	PMP22	-0.609	3.32E-05
**Down**	LYVE1	-0.609	1.39E-03
**Down**	LTBP2	-0.608	2.00E-03
**Down**	COL4A2	-0.608	2.65E-03
**Down**	IFI30	-0.608	9.46E-04
**Down**	C3	-0.608	1.12E-02
**Down**	C10ORF10	-0.608	5.45E-04
**Down**	IBSP	-0.606	4.49E-03
**Down**	CLIC1	-0.605	1.33E-07
**Down**	CCN1	-0.604	9.31E-03
**Down**	VAMP5	-0.604	2.61E-06
**Down**	FMOD	-0.604	9.70E-03
**Down**	IGFBP6	-0.603	1.30E-02
**Down**	FNDC3B	-0.603	4.89E-05
**Down**	DPY19L1	-0.603	5.76E-05
**Down**	S100A6	-0.603	1.51E-06
**Down**	ITGA3	-0.601	4.59E-05
**Down**	DUSP4	-0.599	9.61E-04

**Down**	FLNA	-0.598	6.25E-05
**Down**	PDIA5	-0.598	7.32E-05
**Down**	MT1X	-0.598	2.15E-03
**Down**	CRISPLD2	-0.598	1.52E-03
**Down**	GFPT2	-0.596	8.77E-03
**Down**	PPL	-0.593	1.52E-03
**Down**	SOCS2	-0.593	1.84E-02
**Down**	FCGR2A	-0.591	7.52E-04
**Down**	OSBPL3	-0.590	4.60E-05
**Down**	ADAMTS1	-0.589	2.92E-03
**Down**	RARRES1	-0.589	1.43E-02
**Down**	IRS2	-0.588	3.12E-04
**Down**	CDKN1A	-0.587	2.45E-04
**Down**	GPRC5A	-0.587	7.02E-03
**Down**	ITGA2	-0.587	1.79E-03
**Down**	EGFR	-0.587	9.17E-02
**Down**	LTBP3	-0.586	1.25E-04
**Down**	FKBP11	-0.585	2.42E-04

Supplementary Table 2. Clinical information of glioma patients in TMA slides (TMA1573, 2248, 2249, 2758).

Parameter		No. (N = 182)	%
Gender	Male	100	54.9
	Female	82	45.1
Mean age (at onset)		53.6	
Treatment	Surgery	10	5.5
	Surgery + CCRT	115	63.2
	Surgery + CCRT + GKS	3	1.6
	Surgery + CCRT + CTx	47	25.8
	Surgery + CCRT + CTx + GKS	6	3.3
	not known	1	0.5
Tumor location	Frontal	83	45.6
	Temporal	54	29.7
	Parietal	23	12.6
	Occipital	1	0.5
	Others	21	11.5
ELAVL2 IHC score	0 (<10%)	24	13.2
	1+ (10-25%)	60	33.0
	2+ (25-50%)	41	22.5
	3+ (50-75%)	44	24.2
	4+ (>75%)	13	7.1

Histology	Total	LOH 1p/19q	IDH1 mutation	MGMT methylation	EGFR amplification
WHO grade II	5 (2.7%)	4	5	4	0
WHO grade III	11 (6.1%)	6	10	6	0
WHO grade IV	166 (91.2%)	6	18	88	45
Total	182 (100%)	16	33	98	45

Supplementary Table 3. List of positively correlated genes with ELAVL2 in TCGA, GSE16011, and GSE53733 datasets (R correlation p-value <= 0.0001, FDR applied). Only top 200 are shown.

TCGA			GSE16011			GSE53733		
hugo	r-value	r-pvalue	hugo	r-value	r-pvalue	hugo	r-value	r-pvalue
ELAVL2	1.00	0	ELAVL2	1.00	0	ELAVL2	1.00	0
HMP19	0.51	1.47E-29	TAGLN3	0.64	7.40E-16	SHANK2	0.78	2.98E-11
PAK7	0.50	1.15E-28	HMP19	0.64	5.57E-16	ATRNL1	0.72	1.19E-08
ELAVL4	0.50	3.34E-28	KIAA1107	0.64	8.86E-16	PAK3	0.72	1.30E-08
TMEM35	0.49	1.53E-27	STXBP1	0.63	1.22E-15	SRRM4	0.68	1.44E-07
PAK3	0.47	1.59E-25	TMEM35	0.63	1.39E-15	PHACTR1	0.68	1.54E-07
CDK5R1	0.47	1.29E-24	INA	0.62	1.10E-14	PLCXD2	0.68	1.67E-07
INA	0.46	8.65E-24	SH3GL2	0.61	1.62E-14	DYNC1I1	0.68	1.77E-07
RUNDC3A	0.46	1.16E-23	ELAVL4	0.61	1.58E-14	RAB3C	0.68	1.87E-07
SHANK2	0.45	2.81E-23	GABRG2	0.61	1.98E-14	RUNDC3A	0.68	2.01E-07
PTBP2	0.45	3.17E-23	PAK3	0.61	1.91E-14	NRXN3	0.67	2.20E-07
FGF9	0.45	4.73E-23	SCAI	0.61	3.08E-14	BASP1	0.68	2.26E-07
DNM3	0.45	7.09E-23	CPEB3	0.61	3.60E-14	KCNQ5	0.68	2.36E-07
RALYL	0.45	1.79E-22	SYT13	0.61	3.46E-14	KIAA1107	0.67	2.61E-07
STMN2	0.45	2.45E-22	STXBP5	0.60	5.82E-14	MYT1L	0.67	2.73E-07
DCX	0.44	3.48E-22	CHGA	0.60	6.26E-14	SNAP91	0.66	3.82E-07
TAGLN3	0.44	4.32E-22	MIR124-2HG	0.60	7.36E-14	KSR2	0.66	3.82E-07
OPCML	0.44	1.32E-21	SYNPR	0.60	1.36E-13	PRKCZ	0.66	3.82E-07
WASF1	0.44	2.37E-21	MYT1L	0.59	1.53E-13	CLGN	0.66	3.89E-07
SNAP91	0.43	3.56E-21	SRRM4	0.59	2.39E-13	CELF5	0.66	3.92E-07
ZNF238	0.43	5.97E-21	SH3BGRL2	0.58	9.34E-13	UBE2QL1	0.66	3.97E-07
CAMTA1	0.43	6.95E-21	CELF5	0.58	1.02E-12	CNTNAP2	0.66	4.43E-07
CDH18	0.43	1.77E-20	CDK5R1	0.58	9.91E-13	HECW1	0.66	4.62E-07
SLC17A6	0.43	2.72E-20	FGF13	0.58	1.10E-12	FGF13	0.66	4.76E-07
GNG4	0.42	3.12E-20	KCNJ3	0.57	1.78E-12	PAK7	0.66	5.08E-07
ACTL6B	0.42	3.80E-20	SLC17A6	0.57	3.39E-12	STMN2	0.65	5.61E-07
MAGI1	0.42	3.91E-20	KIF5C	0.57	4.61E-12	TAGLN3	0.65	5.75E-07
DUSP26	0.42	4.75E-20	FAM126B	0.57	4.68E-12	SCN3B	0.65	6.36E-07
KIAA1107	0.42	6.60E-20	RPS6KA5	0.57	5.04E-12	PCSK2	0.65	6.44E-07
GNAO1	0.42	6.43E-20	DACH2	0.56	5.17E-12	PEX5L	0.65	6.53E-07
MYT1L	0.42	8.31E-20	CRTAC1	0.56	5.87E-12	SH3GL2	0.65	6.77E-07
NRXN1	0.42	8.54E-20	STMN2	0.56	5.97E-12	CRTAC1	0.65	7.62E-07
SH3GL2	0.42	9.08E-20	SNAP25	0.56	7.13E-12	PGBD5	0.65	7.90E-07
SEC61A2	0.42	1.44E-19	RUNDC3A	0.56	9.57E-12	GABRB3	0.65	8.03E-07
ZNF804A	0.41	3.24E-19	DYNC1I1	0.56	1.00E-11	ATCAY	0.64	8.35E-07
SNAP25	0.41	3.37E-19	KCTD16	0.56	1.10E-11	NAP1L3	0.64	9.32E-07
PCSK2	0.41	3.47E-19	ATP8A2	0.56	1.35E-11	ST8SIA3	0.64	9.46E-07
TSPYL4	0.41	3.85E-19	FAM117B	0.55	1.52E-11	ANO5	0.64	1.03E-06
LPPR1	0.41	4.16E-19	CPLX1	0.55	1.56E-11	NMNAT2	0.64	1.07E-06
KCNB1	0.41	4.58E-19	MAST1	0.55	1.65E-11	SYNPR	0.64	1.17E-06

CUX2	0.41	6.12E-19	WASF1	0.55	1.92E-11	STXBP1	0.64	1.37E-06
RIMS3	0.41	6.58E-19	CHGB	0.55	1.92E-11	SRCIN1	0.64	1.37E-06
ATP1A3	0.41	6.45E-19	RAB3C	0.55	2.22E-11	DUSP26	0.63	1.47E-06
TMEM151B	0.41	7.88E-19	NMNAT2	0.55	2.58E-11	ELAVL4	0.63	1.47E-06
ATP8A1	0.41	9.64E-19	BASP1	0.55	3.23E-11	MTURN	0.63	1.53E-06
GAD1	0.41	9.71E-19	AKAP6	0.55	3.79E-11	CALN1	0.63	1.55E-06
STXBP1	0.41	9.74E-19	PCSK2	0.55	3.82E-11	FRRS1L	0.63	1.58E-06
STMN4	0.41	1.65E-18	MIR7-3HG	0.55	3.77E-11	SLCO5A1	0.63	1.65E-06
COPG2IT1	0.41	1.72E-18	ATP8A1	0.55	3.80E-11	STXBP5	0.63	1.69E-06
SYT1	0.41	1.88E-18	PLCXD2	0.54	4.25E-11	ADRBK2	0.63	1.81E-06
L1CAM	0.40	2.53E-18	ACVR1C	0.54	4.26E-11	SYT4	0.63	1.89E-06
KIF21B	0.40	3.75E-18	SLC12A5	0.54	4.36E-11	ACVR1C	0.63	1.91E-06
CHGA	0.40	6.79E-18	ACTL6B	0.54	4.63E-11	SPHKAP	0.63	2.06E-06
CELSR3	0.40	7.49E-18	CACNA1E	0.54	6.12E-11	HMP19	0.63	2.15E-06
MYT1	0.40	8.60E-18	ST8SIA3	0.54	6.68E-11	FGF9	0.63	2.17E-06
CAMKV	0.40	9.74E-18	NEGR1	0.54	6.95E-11	SLC1A6	0.62	2.37E-06
RAB33A	0.40	1.05E-17	SEPT3	0.54	8.77E-11	HTR2A	0.62	2.38E-06
IL1RAPL1	0.39	1.90E-17	REPS2	0.54	9.47E-11	TMEM246	0.62	2.41E-06
TOX3	0.39	2.38E-17	COPG2IT1	0.54	9.94E-11	INA	0.62	2.49E-06
ANO3	0.39	2.38E-17	PLEKHM3	0.54	9.97E-11	TMEM151B	0.62	3.04E-06
STMN1	0.39	2.44E-17	RAB3A	0.53	1.14E-10	LINC00632	0.62	3.05E-06
MTSS1	0.39	2.65E-17	FGF9	0.53	1.23E-10	DNM3	0.62	3.09E-06
SATB1	0.39	2.83E-17	LOC285147	0.53	1.26E-10	ELMO1	0.62	3.28E-06
PHACTR1	0.39	4.56E-17	SNAP91	0.53	1.50E-10	RPS6KA5	0.62	3.31E-06
PLCL1	0.39	5.14E-17	SH3GL3	0.53	1.77E-10	PPP1R3F	0.62	3.32E-06
FUT9	0.39	5.50E-17	RNF165	0.53	1.75E-10	PHACTR3	0.62	3.55E-06
BASP1	0.39	6.01E-17	CDH18	0.53	2.24E-10	SLC9A6	0.62	3.79E-06
HPCAL4	0.39	6.40E-17	RIMS3	0.53	2.25E-10	GRM1	0.61	3.89E-06
CLGN	0.39	6.59E-17	SLC8A2	0.53	2.24E-10	TMEM35	0.61	3.92E-06
BSN	0.39	6.98E-17	ZNF248	0.53	2.68E-10	RAB33A	0.61	3.92E-06
RGS7	0.39	6.92E-17	CELF3	0.53	2.70E-10	JAKMIP1	0.61	3.99E-06
GDAP1L1	0.39	7.90E-17	UNC5A	0.52	2.83E-10	RAB9B	0.61	4.22E-06
ARHGAP33	0.39	1.27E-16	TTC9B	0.52	2.82E-10	SRRM3	0.61	4.23E-06
CEP170	0.38	1.41E-16	DNM3	0.52	2.85E-10	PFN2	0.61	4.41E-06
RBFOX1	0.38	1.49E-16	LINC00632	0.52	2.82E-10	JPH3	0.61	4.50E-06
GRM5	0.38	1.91E-16	GPR85	0.52	2.96E-10	PPFIA2	0.61	4.53E-06
REEP1	0.38	2.85E-16	TUNAR	0.52	3.51E-10	SYT1	0.61	4.56E-06
ZNF248	0.38	3.04E-16	SVOP	0.52	3.63E-10	PPP2R2C	0.61	4.85E-06
R3HDM1	0.38	3.86E-16	LINC00294	0.52	3.60E-10	WASF1	0.61	4.91E-06
CAMSAP2	0.38	4.23E-16	AFF3	0.52	3.61E-10	CACNA1E	0.61	4.95E-06
PIK3R3	0.38	4.35E-16	CAMKV	0.52	4.22E-10	RIMS2	0.61	4.96E-06
MYCN	0.38	5.28E-16	PAK7	0.52	4.21E-10	FBXL16	0.61	5.13E-06
SCN3B	0.38	7.38E-16	SYN2	0.52	4.39E-10	LINC01105	0.61	5.15E-06
CLASP2	0.38	8.01E-16	LRRC7	0.52	4.49E-10	SH3GL3	0.61	5.18E-06

CCP110	0.38	8.72E-16	SYT4	0.52	5.11E-10	CPLX1	0.60	5.91E-06
GSTA4	0.38	9.33E-16	UBE2QL1	0.52	5.08E-10	SYT13	0.60	6.00E-06
PPP3CB	0.37	1.21E-15	SRCIN1	0.52	5.69E-10	ATL1	0.60	6.44E-06
TUBB4A	0.37	1.84E-15	PPP3CB	0.52	5.93E-10	PHYHIP	0.60	6.65E-06
LOC284244	0.37	2.00E-15	MAPK8	0.51	7.53E-10	AMPH	0.60	7.43E-06
UBQLN2	0.37	1.98E-15	CUX2	0.51	7.48E-10	SH3BGRL2	0.60	7.83E-06
MARCKSL1	0.37	2.02E-15	PRKCE	0.51	7.99E-10	BEND4	0.60	8.07E-06
DGKB	0.37	2.43E-15	RBFOX1	0.51	8.11E-10	VAT1L	0.60	8.13E-06
C11ORF95	0.37	3.89E-15	CBLN2	0.51	9.01E-10	RNF165	0.60	8.21E-06
KIF5C	0.37	4.42E-15	ATP2B3	0.51	1.01E-09	REPS2	0.60	8.21E-06
CHGB	0.37	4.72E-15	GNAI1	0.51	1.12E-09	LRCH2	0.60	8.57E-06
SYN1	0.37	4.92E-15	STXBP6	0.51	1.13E-09	SMPD3	0.60	8.66E-06
KCND2	0.37	5.21E-15	RAB33A	0.51	1.20E-09	NPY	0.60	8.67E-06
DYNC1I1	0.37	5.40E-15	KIF21B	0.51	1.22E-09	SHISA7	0.60	9.04E-06
CA10	0.37	5.73E-15	GDPD1	0.51	1.23E-09	KLHL32	0.59	9.32E-06
RPRM	0.37	5.70E-15	RIMS1	0.51	1.30E-09	STMN4	0.59	9.35E-06
GABRG2	0.37	6.36E-15	RIMS2	0.51	1.32E-09	KIF21B	0.59	9.44E-06
NRXN2	0.37	6.33E-15	DUSP26	0.51	1.35E-09	ATP8A1	0.59	9.51E-06
AMPH	0.37	6.97E-15	RTN1	0.51	1.43E-09	RIMBP2	0.59	9.75E-06
TMEM246	0.36	7.00E-15	CPLX2	0.51	1.50E-09	DSCAML1	0.59	1.05E-05
SOX4	0.36	6.98E-15	ELFN2	0.51	1.65E-09	KCNJ3	0.59	1.08E-05
RAPGEF4	0.36	7.87E-15	PHACTR3	0.50	1.85E-09	NECAB2	0.59	1.09E-05
SH3GL3	0.36	8.35E-15	WSCD2	0.50	1.85E-09	CHGA	0.59	1.12E-05
RNF144A	0.36	8.90E-15	SLCO5A1	0.50	1.90E-09	ATP1A3	0.59	1.14E-05
CXXC4	0.36	9.01E-15	FRRS1L	0.50	1.93E-09	SORCS1	0.59	1.15E-05
FLRT1	0.36	9.38E-15	USP49	0.50	1.95E-09	TMEM196	0.59	1.16E-05
KIF1A	0.36	9.86E-15	HECW1	0.50	2.26E-09	AFF3	0.59	1.17E-05
PPP1R1A	0.36	9.84E-15	JPH3	0.50	2.38E-09	BRSK2	0.59	1.18E-05
RALGPS1	0.36	1.12E-14	DDN	0.50	2.50E-09	ERC2	0.59	1.18E-05
GNG3	0.36	1.54E-14	ATP6V1G2	0.50	2.63E-09	GFRA1	0.59	1.19E-05
SCN2A	0.36	2.46E-14	ACVR2A	0.50	2.75E-09	RAB11FIP4	0.59	1.32E-05
KLRC3	0.36	2.56E-14	KSR2	0.50	3.00E-09	KCTD16	0.59	1.34E-05
HPCA	0.36	2.56E-14	RNF41	0.50	3.07E-09	TCEAL2	0.59	1.35E-05
PJA1	0.36	3.08E-14	SATB1	0.50	3.05E-09	RASL10A	0.59	1.36E-05
SLC12A5	0.36	3.24E-14	BRSK2	0.50	3.04E-09	GDAP1	0.59	1.36E-05
NAP1L3	0.36	3.27E-14	FSD1	0.50	3.20E-09	FAM117B	0.58	1.44E-05
PTPRT	0.36	3.56E-14	ZDHHC22	0.50	3.21E-09	CA10	0.58	1.49E-05
ARHGEF9	0.35	4.67E-14	RIPPLY2	0.50	3.60E-09	CACNA2D2	0.58	1.50E-05
SMPD3	0.35	5.70E-14	IGFBPL1	0.50	3.58E-09	CDH18	0.58	1.62E-05
DLG3	0.35	6.77E-14	ASXL3	0.50	4.03E-09	PKIA	0.58	1.64E-05
GPSM2	0.35	7.19E-14	ARHGEF9	0.50	4.01E-09	RPRM	0.58	1.90E-05
PRKCZ	0.35	7.28E-14	MOAP1	0.49	4.33E-09	CELF4	0.58	1.91E-05
PCDH7	0.35	7.87E-14	TMEM132D	0.49	4.46E-09	NKAIN1	0.58	1.91E-05
TMEM257	0.35	8.25E-14	ATP1A3	0.49	5.00E-09	SPOCK3	0.58	1.92E-05

NEFM	0.35	1.05E-13	NAP1L3	0.49	5.21E-09	SLITRK1	0.58	1.92E-05
MLLT11	0.35	1.16E-13	GABRB3	0.49	5.29E-09	ZBTB18	0.58	1.93E-05
PLCB4	0.35	1.21E-13	NAPB	0.49	5.32E-09	LOC283588	0.58	2.10E-05
GABBR1	0.35	1.21E-13	ATL1	0.49	5.36E-09	KCNH8	0.58	2.24E-05
KLHL9	0.35	1.42E-13	KIF3A	0.49	5.51E-09	SNAP25	0.58	2.26E-05
RFPL1-AS1	0.35	1.48E-13	GSTA4	0.49	5.70E-09	CCP110	0.58	2.27E-05
YPEL1	0.35	1.64E-13	AMN1	0.49	5.68E-09	GNG4	0.57	2.33E-05
CLSTN2	0.35	1.84E-13	TUB	0.49	5.80E-09	SEPT3	0.57	2.34E-05
B3GALT2	0.35	2.17E-13	PRKCZ	0.49	5.99E-09	CAMKV	0.57	2.52E-05
ZFPM2	0.35	2.39E-13	GNG4	0.49	6.93E-09	FRMD4A	0.57	2.54E-05
HDGFRP3	0.35	2.46E-13	LINC01105	0.49	7.09E-09	RGS7	0.57	2.56E-05
KIAA1644	0.35	2.59E-13	PLPPR1	0.49	7.26E-09	RBFOX1	0.57	2.61E-05
ATAT1	0.35	2.58E-13	LOC283588	0.49	7.37E-09	CHGB	0.57	2.71E-05
EYA1	0.35	2.59E-13	ZNF25	0.49	7.48E-09	KIF5C	0.57	2.72E-05
DGKI	0.34	2.82E-13	CCDC85A	0.49	7.45E-09	NOL4	0.57	2.72E-05
MTMR9	0.34	2.88E-13	AMER3	0.49	7.82E-09	PDZRN4	0.57	2.94E-05
ATP2B2	0.34	3.46E-13	NEUROD6	0.49	7.84E-09	CCNG2	0.57	3.09E-05
RNFT2	0.34	3.46E-13	UNC13C	0.49	8.07E-09	LRRC8B	0.57	3.10E-05
JPH3	0.34	3.46E-13	CALN1	0.49	9.18E-09	CDK5R1	0.57	3.26E-05
RIMS2	0.34	4.47E-13	ADAM22	0.48	9.24E-09	DCX	0.57	3.34E-05
PDE10A	0.34	4.50E-13	RAB11FIP4	0.48	9.92E-09	DACH2	0.57	3.37E-05
THSD7A	0.34	5.29E-13	DOK6	0.48	9.95E-09	CCDC85A	0.57	3.39E-05
FGF13	0.34	5.39E-13	RALGPS1	0.48	1.00E-08	GABRG2	0.57	3.58E-05
ASRGL1	0.34	6.15E-13	KAT6B	0.48	1.07E-08	MBP	0.56	3.68E-05
FAM155A	0.34	6.49E-13	PCLO	0.48	1.07E-08	RAP2A	0.56	3.79E-05
CDC7	0.34	6.99E-13	SLC8A3	0.48	1.09E-08	PPP3CB	0.56	3.91E-05
NOL4	0.34	8.49E-13	SNCB	0.48	1.12E-08	TMEFF2	0.56	3.91E-05
SYN2	0.34	1.13E-12	RGS7	0.48	1.11E-08	SLC8A2	0.56	3.95E-05
FHOD3	0.34	1.28E-12	BSN	0.48	1.24E-08	WNK2	0.56	3.96E-05
GPR17	0.34	1.27E-12	PACSIN1	0.48	1.35E-08	ELFN2	0.56	3.97E-05
PTPRD	0.34	1.29E-12	C9ORF91	0.48	1.49E-08	PCDH20	0.56	4.05E-05
PLCB1	0.34	1.29E-12	TMEM151B	0.48	1.54E-08	MAGEE1	0.56	4.09E-05
ZNF821	0.34	1.36E-12	PRPSAP2	0.48	1.56E-08	UNC5A	0.56	4.19E-05
TOP2B	0.33	1.50E-12	TTC9	0.48	1.56E-08	PDZD4	0.56	4.21E-05
CELF3	0.33	1.53E-12	SLITRK1	0.48	1.63E-08	VIPR2	0.56	4.60E-05
WDR47	0.33	1.55E-12	WDR47	0.48	1.65E-08	LINC00889	0.56	4.61E-05
DNAJC6	0.33	1.83E-12	MTURN	0.48	1.65E-08	ZNF488	0.56	4.65E-05
MGC12488	0.33	1.87E-12	RBFOX3	0.48	1.70E-08	PCP4	0.56	4.70E-05
OMG	0.33	1.94E-12	GDAP1	0.48	1.73E-08	DLGAP2	0.56	4.78E-05
PDZRN4	0.33	2.06E-12	ANKRD46	0.48	1.76E-08	NGEF	0.56	4.86E-05
PHF16	0.33	2.10E-12	KIRREL3	0.48	1.82E-08	RIMS3	0.56	4.87E-05
ATP6V1G2	0.33	2.10E-12	SCN3B	0.48	1.88E-08	SYN1	0.56	5.10E-05
HOXD3	0.33	2.12E-12	DSCAML1	0.48	2.00E-08	OTUD7A	0.56	5.31E-05
GABRA5	0.33	2.39E-12	VSTM2L	0.48	2.03E-08	RNF41	0.56	5.42E-05

NMNAT2	0.33	2.43E-12	GAD1	0.47	2.12E-08	ATP8A2	0.55	5.68E-05
FAM171A1	0.33	2.43E-12	OLFM3	0.47	2.13E-08	FAM133A	0.55	5.70E-05
RUFY3	0.33	2.60E-12	RALYL	0.47	2.12E-08	ZNF536	0.55	6.02E-05
NECAP1	0.33	2.65E-12	PCP4L1	0.47	2.14E-08	DPP10-AS1	0.55	6.14E-05
USP11	0.33	2.65E-12	ANO3	0.47	2.15E-08	PLCL1	0.55	6.17E-05
NFIB	0.33	2.73E-12	TBPL1	0.47	2.22E-08	ST18	0.55	6.27E-05
CSNK1E	0.33	2.76E-12	KLHL9	0.47	2.29E-08	PCDH7	0.55	6.27E-05
SEZ6L	0.33	2.80E-12	TMEM246	0.47	2.28E-08	FEZF2	0.55	6.28E-05
DLL3	0.33	2.88E-12	IPO5P1	0.47	2.27E-08	LRRTM4	0.55	6.29E-05
EVL	0.33	2.96E-12	CNTN3	0.47	2.41E-08	CNTN3	0.55	6.32E-05
NDRG3	0.33	2.97E-12	SSTR2	0.47	2.47E-08	STX1B	0.55	6.37E-05
SCN3A	0.33	3.02E-12	CSMD3	0.47	2.56E-08	FRMPD4	0.55	6.38E-05
CYFIP2	0.33	3.47E-12	CBX1	0.47	2.75E-08	DLG2	0.55	6.52E-05
RPS6KA5	0.33	3.75E-12	STX1B	0.47	2.75E-08	ANKS1B	0.55	6.75E-05
AKT3	0.33	3.82E-12	CLSTN2	0.47	2.89E-08	FAM19A2	0.55	6.76E-05
C5ORF30	0.33	3.83E-12	RAPGEF4	0.47	2.95E-08	CPLX2	0.55	6.76E-05
SORCS3	0.33	3.84E-12	SLC9A6	0.47	2.96E-08	UBE2E2	0.55	6.80E-05
ADAM22	0.33	3.97E-12	OPCML	0.47	2.99E-08	ZNF804A	0.55	6.82E-05
MVB12B	0.33	4.39E-12	RAB9B	0.47	3.05E-08	KCNIP2	0.55	6.82E-05
CRB1	0.33	4.73E-12	SV2B	0.47	3.11E-08	CSMD3	0.55	7.57E-05
SS18L1	0.33	4.90E-12	JAKMIP1	0.47	3.20E-08	RELN	0.55	7.77E-05
KCNC1	0.33	5.59E-12	TRIM37	0.47	3.22E-08	TMCC2	0.55	8.09E-05
RAP2A	0.33	5.58E-12	ATCAY	0.47	3.71E-08	CD24	0.55	8.10E-05
ACOT7	0.33	5.73E-12	DLG2	0.47	3.85E-08	IGSF21	0.54	8.43E-05
ZNF536	0.33	5.87E-12	LSM11	0.47	3.88E-08	FAM107B	0.54	8.43E-05
CRMP1	0.33	5.96E-12	CAMK1D	0.47	4.07E-08	ANKRD46	0.54	8.46E-05
SLC1A1	0.33	5.96E-12	PDE1A	0.47	4.17E-08	KCNJ9	0.54	8.47E-05
ELMO1	0.33	6.07E-12	SPHKAP	0.46	4.48E-08	SCN2A	0.54	8.63E-05

Supplementary Table 4. List of primers used in RT-qPCR.

Gene	Direction	Sequence (5'-3')
GAPDH	Forward	GTCTCCTCTGACTTCAACAGCG
	Reverse	ACCACCCTGTTGCTGTAGCCAA
ELAVL2	Forward	GCGAATTGAGGCAGAAGAAG
	Reverse	CTGGTACAGCTGGGAAAGGA
Vimentin	Forward	GAGAACTTTGCCGTTGAAGC
	Reverse	CTCAATGTCAAGGGCCATCT
CDH2	Forward	AGGATCAACCCCATACACCA
	Reverse	TGGTTTGACCACGGTGACTA
ZEB1	Forward	GCACAACCAAGTGCAGAAGA
	Reverse	ACTTTGCCTGGTTCAGGAGA
ZEB2	Forward	AATGCACAGAGTGTGGCAAGGC
	Reverse	CTGCTGATGTGCGAACTGTAGG
CD44	Forward	AGGAACAGTGGTTTGGCAAC
	Reverse	GGTGTTGTCCTTCCTTGCAT
MMP3	Forward	TGCTTTGTCCTTTGATGCTG
	Reverse	GGAAGAGATGGCCAAAATGA
MMP9	Forward	CATCGTCATCCAGTTTGGTG
	Reverse	AGGGACCACAACTCGTCATC
TWIST1	Forward	ATCAAACTGGCCTGCAAAAC
	Reverse	TGCATTTTACCATGGGTCCT
TWIST2	Forward	TTTCACGCCGCTATTCTTTT
	Reverse	GAGCAGGATACACAGCCACA
SNAI1	Forward	TGCCCTCAAGATGCACATCCGA
	Reverse	GGGACAGGAGAAGGGCTTCTC
SNAI2	Forward	ATCTGCGGCAAGGCGTTTTCCA
	Reverse	GAGCCCTCAGATTTGACCTGTC
SERPINE1	Forward	TCATGGACAGACCCTTCCTC
	Reverse	ATGGCAATGTGACTGGAACA
VIPR2	Forward	TGCACCAGGAATTACATCCA
	Reverse	ACCAGCACTGAGATGGCTCT
SLC8A2	Forward	GGAGATCACCATCACCAAGG
	Reverse	GAGGTTGGACACCGTCTCAT
RPRM	Forward	CCCTCTGAAAGTCCTCATGC
	Reverse	CCCCGCATTCCAAGTAAGTA
CDH18	Forward	CGGAGAGGAAGACACAGAGG
	Reverse	TCCTCAGCAGCAGAAGGATT
HMP19	Forward	TACGGTCACCATCCTTGTCA
	Reverse	GGCTTTGTAAACCACCAGGA
PLCL1	Forward	TTTTGGAATTGTGGCTGTCA
	Reverse	CGTGTGAAGGTCCATCATTG
CRTAC1	Forward	AGGGCTTCAACAACAACTGG
	Reverse	GCCCCACTCTTCTTGGTGTA
JPH3	Forward	AATCCTTGCCTGTCGCTCTA
	Reverse	TTGAGCTCATCCCCATTCTC
DUSP26	Forward	GGCAGCTAGGCTGTAGATGG
	Reverse	GGTGAGGGAGAGAGGGAAAC
SH3GL3	Forward	TTTGAGCCAGAAAACCAAGG
	Reverse	GAATCCCGATTCTCCGTGTA
DNM3	Forward	AAATCAGGTGATTCGCAAGG
	Reverse	AGGACAAGCTTTCCGCAGTA
BASP1	Forward	CAATGCCAATCCTCCATTCT
	Reverse	AACTACAGGTGCACCCAACC
REPS2	Forward	ACATGGCTGACCTTCCTGTC
	Reverse	CCTCAGACACGTCCTTTGGT
ELAVL1	Forward	TGTTCTCTCGGTTTGGGCGGAT
	Reverse	TCTTCTGCCTCCGACCGTTTGT
ELAVL3	Forward	TGCAGACAAAGCCATCAACACCC
	Reverse	GCTGACGTACAGGTTAGCATCC
ELAVL4	Forward	CCCAGAAGGAACTGGAGCAACT
	Reverse	CCTTTGATGGCTTCTTCTGCCTC

Supplementary Table 5. List of siRNAs.

siRNA		Duplex Sequence (5'-3')
Negative Control	Sense	CCUCGUGCCGUUCCAUCAGGUAGUU
	Antisense	CUACCUGAUGGAACGGCACGAGGUU
ELAVL2 #1	Sense	GACAGAGUACUGCAGGUCU
	Antisense	AGACCUGCAGUACUCUGUC
ELAVL2 #2	Sense	CUGUCUAAUGGGCCAACUU
	Antisense	AAGUUGGCCCAUUAGACAG
ELAVL2 #3	Sense	GAGGAACUAAAGAGUCUCU
	Antisense	AGAGACUCUUUAGUUCCUC
SH3GL3 #1	Sense	CUGAACACUGUGUCGAAGA
	Antisense	UCUUCGACACAGUGUUCAG
SH3GL3 #2	Sense	GUAAGAUACCAGACGAAGA
	Antisense	UCUUCGUCUGGUAUCUUAC
DNM3 #1	Sense	GUCUGAGAACCAUUGGAGU
	Antisense	ACUCCAAUGGUUCUCAGAC
DNM3 #2	Sense	GAUUGUUGCUAACCACAUU
	Antisense	AAUGUGGUUAGCAACAAUC
METTL3 #1	Sense	AUAGUCACAGAAUUCUUGCTT
	Antisense	GUUGAAAGGCAUUGAGAAUCU
