# Supplementary material for: ELAVL2 loss promotes aggressive mesenchymal transition in glioblastoma
Source: NPJ Precis Oncol. 2024 Mar 28;8:79. doi: 10.1038/s41698-024-00566-1 (PMC10978835; doi:10.1038/s41698-024-00566-1)
Supplement: Supplementary file 2 — REPORTING SUMMARY [file 41698_2024_566_MOESM2_ESM.pdf]

nature portfolio

Corresponding author(s): DAPR NPJPRECISIONONCOLOGY-03592R

Last updated by author(s): Oct 6, 2023

Reporting Summary

Nature Portfolio wishes to improve the reproducibility of the work that we publish. This form provides structure for consistency and transparency in reporting. For further information on Nature Portfolio policies, see our Editorial Policies and the Editorial Policy Checklist.

Statistics

For all statistical analyses, confirm that the following items are present in the figure legend, table legend, main text, or Methods section.

n/a	Confirmed	
☐	☒	The exact sample size (*n*) for each experimental group/condition, given as a discrete number and unit of measurement
☐	☒	A statement on whether measurements were taken from distinct samples or whether the same sample was measured repeatedly
☐	☒	The statistical test(s) used AND whether they are one- or two-sided<br>*Only common tests should be described solely by name; describe more complex techniques in the Methods section.*
☐	☒	A description of all covariates tested
☐	☒	A description of any assumptions or corrections, such as tests of normality and adjustment for multiple comparisons
☐	☒	A full description of the statistical parameters including central tendency (e.g. means) or other basic estimates (e.g. regression coefficient) AND variation (e.g. standard deviation) or associated estimates of uncertainty (e.g. confidence intervals)
☐	☒	For null hypothesis testing, the test statistic (e.g. *F*, *t*, *r*) with confidence intervals, effect sizes, degrees of freedom and *P* value noted<br>*Give P values as exact values whenever suitable.*
☒	☐	For Bayesian analysis, information on the choice of priors and Markov chain Monte Carlo settings
☒	☐	For hierarchical and complex designs, identification of the appropriate level for tests and full reporting of outcomes
☒	☐	Estimates of effect sizes (e.g. Cohen's *d*, Pearson's *r*), indicating how they were calculated

Our web collection on statistics for biologists contains articles on many of the points above.

Software and code

Policy information about availability of computer code

Data collection	Gene alteration frequency and copy number data of ELAVL family members in TCGA glioma patients were obtained from cBioPortal online platform (https://www.cbioportal.org). Pan-cancer dataset of whole genomes in cBioPortal was also used to assess the ELAVL2 alteration frequency across various cancer datasets. Following gene expression datasets and clinical information of glioma patients were obtained from GlioVis (http://gliovis.bioinfo.cnio.es) to analyze ELAVL2 mRNA expression levels at various features of glioma: TCGA GBMLGG, CGGA, Ivy GBM, and TCGA U133a. Additionally, GSE53733 and GSE16011 datasets were acquired from the Gene Expression Omnibus (GEO) database.
Data analysis	1. R/Bioconductor programming language (3.6.3) was used to perform the following bioinformatic analyses and generate corresponding plots.<br>2. Pathway analysis on ELAVL2-high and -low groups was performed using the parametric analysis of gene set enrichment (PAGE), which is implemented in PGSEA R package (Kim and Volsky, 2005).<br>3. Gene set enrichment analysis (GSEA) (www.broadinstitute.org) was performed on the desktop version of GSEA (v4.2.0) (Subramanian et al., 2005).<br>4. The weighted gene co-expression network analysis (WGCNA) was performed using the WGCNA R package (Langfelder and Horvath, 2008).<br>5. Gene correlation analyses were carried out on R2 Genomics Analysis and Visualization Platform (http://r2.amc.nl) at R correlation p-value ≤ 0.0001.<br>6. Differential gene expression analysis was performed using the limma R package (Ritchie et al., 2015)<br>7. Clusterprofiler R package was utilized to conduct functional enrichment analysis on the differentially expressed gene (DEG) profile of the ELAVL2-high GBM patients (Wu et al., 2021).<br>8. The core analysis of Ingenuity Pathway Analysis (IPA; QIAGEN) was used to conduct additional functional enrichment analysis (https://digitalinsights.qiagen.com/).<br>9. Kaplan-Meier survival analysis using the log-rank statistic was conducted employing the 'surv_cutpoint()' function from the survminer R package.

10. Statistical analyses were performed using GraphPad Prism (9.0), IBM SPSS Statistics (22.0), and R/Bioconductor programming language (3.6.3).
11. Immunoblotting images were analyzed using Image Lab software (BIO-RAD).

For manuscripts utilizing custom algorithms or software that are central to the research but not yet described in published literature, software must be made available to editors and reviewers. We strongly encourage code deposition in a community repository (e.g. GitHub). See the Nature Portfolio guidelines for submitting code & software for further information.

Data

Policy information about availability of data

All manuscripts must include a data availability statement. This statement should provide the following information, where applicable:

- Accession codes, unique identifiers, or web links for publicly available datasets
- A description of any restrictions on data availability
- For clinical datasets or third party data, please ensure that the statement adheres to our policy

TCGA-GBM and -LGG datasets used in this study are publicly available in the National Cancer Institute Genomic Data Commons (GDC) Data Portal (https://portal.gdc.cancer.gov/) repository, cBioPortal (https://www.cbioportal.org) and GlioVis (http://gliovis.bioinfo.cnio.es) online platforms. Other public GBM datasets used are available through the GEO under the accession codes GSE53733 and GSE16011.

Research involving human participants, their data, or biological material

Policy information about studies with human participants or human data. See also policy information about sex, gender (identity/presentation), and sexual orientation and race, ethnicity and racism.

Reporting on sex and gender	Gender information of the deceased glioma patients on tissue microarray is included in the supplementary information. Findings do not apply to only one sex or gender. Sex and gender information were not considered in study design.
Reporting on race, ethnicity, or other socially relevant groupings	N/A
Population characteristics	N/A
Recruitment	N/A
Ethics oversight	Seoul National University Hospital

Note that full information on the approval of the study protocol must also be provided in the manuscript.

Field-specific reporting

Please select the one below that is the best fit for your research. If you are not sure, read the appropriate sections before making your selection.

☒ Life sciences ☐ Behavioural & social sciences ☐ Ecological, evolutionary & environmental sciences

For a reference copy of the document with all sections, see nature.com/documents/nr-reporting-summary-flat.pdf

Life sciences study design

All studies must disclose on these points even when the disclosure is negative.

Sample size	Sample size was determined based on similar studies in this field.
Data exclusions	No data were excluded from the analyses
Replication	To ensure robust reproducibility, all in-vitro data presented in this study were repeated three times. Immunoblotting was repeated at least three times to ensure the expression level of a molecule. All cell images (e.g., invasion assay, immunocytochemistry) presented were taken at least three times to obtain valid results. Results from all technical- and biological replicates were consistent among them.
Randomization	Randomization was not applicable to our study since we used established glioblastoma cell lines, and their allocation to experimental groups was based on specific criteria, such as ELAVL2 expression status.
Blinding	The investigators were blinded to the clinical information of glioma patients on the tissue microarray while scoring the staining intensity of ELAVL2 on a scale from 0 to 4+ based on the percentage of stained cells to ensure impartiality.

Reporting for specific materials, systems and methods

We require information from authors about some types of materials, experimental systems and methods used in many studies. Here, indicate whether each material, system or method listed is relevant to your study. If you are not sure if a list item applies to your research, read the appropriate section before selecting a response.

Materials & experimental systems

n/a	Involved in the study
☐	☒ Antibodies
☐	☒ Eukaryotic cell lines
☒	☐ Palaeontology and archaeology
☒	☐ Animals and other organisms
☒	☐ Clinical data
☒	☐ Dual use research of concern
☒	☐ Plants

Methods

n/a	Involved in the study
☒	☐ ChIP-seq
☒	☐ Flow cytometry
☒	☐ MRI-based neuroimaging

Antibodies

Antibodies used

1. anti-ELAVL2, Thermo Fisher Cat#PA5-36157, RRID: AB_2553400, Lot#WK3427302
2. anti-ZEB1, Abcam Cat#ab124512, RRID: AB_10971375, Lot#GR3247043-7
3. anti-N-cadherin, Abcam Cat#ab18203, RRID: AB_444317, Lot#GR3404600-1
4. anti-MMP3, Abcam Cat#ab53015, RRID: AB_881242
5. anti-CD44, Abcam Cat#ab157107, RRID: AB_2847859
6. anti-αSMA, Abcam Cat#ab5694, RRID: AB_2223021, Lot#GR234802-1
7. anti-IL6, Thermo Fisher Cat#PA5-118007, RRID: AB_2902612
8. anti-SERPINE1, Thermo Fisher Cat#MA1-40224, RRID: AB_2186871
9. anti-CCN2, Thermo Fisher Cat#PA5-32193, RRID: AB_2549666
10. anti-SH3GL3, ATLAS ANTIBODIES Cat#HPA039381, RRID: AB_10794635, Lot#000027142
11. anti-DNM3, LSBio Cat#LS-C409118, RRID: n/a, Lot#171536
12. β-actin, Santa Cruz Biotechnology Cat#sc-81178, RRID: AB_2223230, Lot#12822
13. anti-Rabbit IgG (H+L), made in goat, Vector Laboratories Cat#PI-1000, RRID: AB_2336198, Lot#ZH0309
14. anti-Mouse IgG (H+L), made in horse, Vector Laboratories Cat#PI-2000, RRID: AB_2336177, Lot#ZH0513
15. anti-E-cadherin, Thermo Fisher Cat#14-3249-82, RRID: AB_1210458, Lot#2416068
16. anti-Vimentin, Abcam Cat#ab137321, RRID: AB_2921312, Lot#GR3390147-3
17. Anti-Rat IgG (H+L) Cross-Adsorbed Secondary Antibody, Alexa Fluor 488, Thermo Fisher Cat#A-11006, RRID: AB_2534074, Lot#2247986
18. Anti-Rabbit IgG (H+L) Cross-Adsorbed Secondary Antibody, Alexa Fluor 594, Thermo Fisher Cat# A-11012, RRID: AB_2534079, Lot#2307302

Validation

1. https://www.thermofisher.com/antibody/product/ELAVL2-Antibody-Polyclonal/PA5-36157
2. https://www.abcam.com/products/primary-antibodies/zeb1-antibody-ab124512.html
3. https://www.abcam.com/products/primary-antibodies/n-cadherin-antibody-intercellular-junction-marker-ab18203.html?productWallTab=ShowAll
4. https://www.abcam.com/products/primary-antibodies/mmp3-antibody-ab53015.html?productWallTab=ShowAll
5. https://www.abcam.com/products/primary-antibodies/cd44-antibody-ab157107.html?productWallTab=ShowAll
6. https://www.abcam.com/products/primary-antibodies/alpha-smooth-muscle-actin-antibody-ab5694.html?productWallTab=ShowAll
7. https://www.thermofisher.com/antibody/product/PA5-118007.html?CID=AFLCA-PA5-118007
8. https://www.thermofisher.com/antibody/product/PAI1-Antibody-clone-MA-33H1F7-Monoclonal/MA1-40224
9. https://www.thermofisher.com/antibody/product/CTGF-Antibody-Polyclonal/PA5-32193
10. https://www.atlasantibodies.com/products/antibodies/primary-antibodies/triple-a-polyclonals/sh3gl3-antibody-hpa039381/
11. https://www.lsbio.com/antibodies/dnm3-antibody-dynamin-3-antibody-ihc-ip-wb-western-ls-c409118/421483
12. https://www.scbt.com/ko/p/beta-actin-antibody-actbd11b7
13. https://vectorlabs.com/products/peroxidase-goat-anti-rabbit-igg/
14. https://vectorlabs.com/products/peroxidase-horse-anti-mouse-igg/
15. https://www.thermofisher.com/antibody/product/CD324-E-Cadherin-Antibody-clone-DECMA-1-Monoclonal/14-3249-82
16. https://www.abcam.com/products/primary-antibodies/vimentin-antibody-ab137321.html?productWallTab=ShowAll
17. https://www.thermofisher.com/antibody/product/Goat-anti-Rat-IgG-H-L-Cross-Adsorbed-Secondary-Antibody-Polyclonal/A-11006
18. https://www.thermofisher.com/antibody/product/Goat-anti-Rabbit-IgG-H-L-Cross-Adsorbed-Secondary-Antibody-Polyclonal/A-11012

Eukaryotic cell lines

Policy information about cell lines and Sex and Gender in Research

Cell line source(s)

Below is a list of primary glioblastoma cell lines with the source and sex of the patients
GBM14: Department of Neurosurgery, SNUH, male
GBM15: Department of Neurosurgery, SNUH, female
GBM28: Department of Neurosurgery, SNUH, female
GBM30: Department of Neurosurgery, SNUH, female
GBM37: Department of Neurosurgery, SNUH, female

Below is a list of commercial cancer cell lines used and the source
SNU407: Korean Cell Line Bank, Cat#00407
HCT8: Korean Cell Line Bank, Cat#10244
HCT15: Korean Cell Line Bank, Cat#10225
SW480: Korean Cell Line Bank, Cat#10228
SW620: Korean Cell Line Bank, Cat#60068
Capan2: Korean Cell Line Bank, Cat#30080
HepG2: Korean Cell Line Bank, Cat#88065
T98G: Laboratory of Sun Ha Paek
U87MG: Laboratory of Sun Ha Paek
U373MG: Laboratory of Sun Ha Paek
U251MG: Laboratory of Sun Ha Paek

Authentication	We utilized primary glioblastoma cell lines derived from patient tumor samples. As these cell lines underwent several culture passages, we conducted extensive genetic testing for key glioblastoma markers, including GFAP, EGFR, MGMT methylation status, 1p/19q deletion status, and IDH1/2 mutation status to validate their glioblastoma identity and ensure their fidelity. Subsequently, the primary cell lines were considered to retain the molecular characteristics of the original patient tumor tissue.
Mycoplasma contamination	All cell lines used in this study were checked for mycoplasma contamination using mycoplasma detection kit (InvivoGen) and were negative
Commonly misidentified lines (See ICLAC register)	None of the cell lines used in this study is included as misidentified cell line in ICLAC register
